# Estrogen receptor inhibition mediates radiosensitization of ER-positive breast cancer models

**DOI:** 10.1038/s41523-022-00397-y

**Published:** 2022-03-10

**Authors:** Anna R. Michmerhuizen, Lynn M. Lerner, Andrea M. Pesch, Connor Ward, Rachel Schwartz, Kari Wilder-Romans, Meilan Liu, Charles Nino, Kassidy Jungles, Ruth Azaria, Alexa Jelley, Nicole Zambrana Garcia, Alexis Harold, Amanda Zhang, Bryan Wharram, Daniel F. Hayes, James M. Rae, Lori J. Pierce, Corey W. Speers

**Affiliations:** 1 Department of Radiation Oncology, University of Michigan, Ann Arbor, MI USA; 2 Rogel Cancer Center, University of Michigan, Ann Arbor, MI USA; 3 Program in Cellular and Molecular Biology, University of Michigan, Ann Arbor, MI USA; 4 Department of Pharmacology, University of Michigan, Ann Arbor, MI USA; 5 Department of Internal Medicine, University of Michigan, Ann Arbor, MI USA

**Keywords:** Targeted therapies, Breast cancer, Non-homologous-end joining, Radiotherapy, Breast cancer

## Abstract

Endocrine therapy (ET) is an effective first-line therapy for women with estrogen receptor-positive (ER + ) breast cancers. While both ionizing radiation (RT) and ET are used for the treatment of women with ER+ breast cancer, the most effective sequencing of therapy and the effect of ET on tumor radiosensitization remains unclear. Here we sought to understand the effects of inhibiting estrogen receptor (ER) signaling in combination with RT in multiple preclinical ER+ breast cancer models. Clonogenic survival assays were performed using variable pre- and post-treatment conditions to assess radiosensitization with estradiol, estrogen deprivation, tamoxifen, fulvestrant, or AZD9496 in ER+ breast cancer cell lines. Estrogen stimulation was radioprotective (radiation enhancement ratios [rER]: 0.51–0.82). Conversely, when given one hour prior to RT, ER inhibition or estrogen depletion radiosensitized ER+ MCF-7 and T47D cells (tamoxifen rER: 1.50–1.60, fulvestrant rER: 1.76–2.81, AZD9496 rER: 1.33–1.48, estrogen depletion rER: 1.47–1.51). Combination treatment resulted in an increase in double-strand DNA (dsDNA) breaks as a result of inhibition of non-homologous end joining-mediated dsDNA break repair with no effect on homologous recombination. Treatment with tamoxifen or fulvestrant in combination with RT also increased the number of senescent cells but did not affect apoptosis or cell cycle distribution. Using an MCF-7 xenograft model, concurrent treatment with tamoxifen and RT was synergistic and resulted in a significant decrease in tumor volume and a delay in time to tumor doubling without significant toxicity. These findings provide preclinical evidence that concurrent treatment with ET and RT may be an effective radiosensitization strategy.

## Introduction

Invasive breast cancer is the leading cause of cancer deaths in women globally, accounting for 15% of all cancer-related deaths^1^. Breast cancer, however, is a heterogeneous disease, and treatment strategies for breast cancer patients are largely determined based on the presence of molecular drivers, including expression of the estrogen receptor (ER). ER expression is present in 70–80% of breast tumors and has been shown to be a significant driver of breast cancer pathogenesis^2^. Based on multiple randomized studies demonstrating its benefit for treatment and prevention, endocrine therapy (ET), which targets ER and downstream ER signaling, is the first-line treatment for women with ER-positive (ER+ ) breast cancer^3^. These therapies include selective estrogen receptor modulators (SERMs), such as tamoxifen, raloxifene, and toremifene, which can act as an ERα partial agonist or antagonist depending on the target tissue^4,5^. Selective estrogen receptor degraders (SERDs), such as fulvestrant, and investigational SERDS including AZD9496, AZD9833, LY3484356, GDC-0810, GDC-0927, GDC-9545, and SAR439859, inhibit ER-mediated cellular proliferation through degradation of ERα^6^. In contrast, aromatase inhibitors (AIs), such as anastrozole, letrozole, and exemestane, are used to block the production of estrogens through inhibition of CYP19 aromatase thereby blocking downstream ER signaling^7^.

Ionizing radiation has also been shown to significantly increase overall survival and decrease rates of locoregional recurrence in ER+ breast cancer patients following breast-conserving surgery and mastectomy^8^. Despite tumor heterogeneity and potential differences in the intrinsic radiation sensitivities of each tumor, all breast cancer patients receive similar scheduling and dosing of radiation without personalization based on molecular characteristics. While ER+ patients receive targeted therapies, including SERMs, SERDs, and AIs, the effects of these therapies on tumor radiosensitization remain unclear. Previous retrospective clinical studies suggest that concurrent administration of tamoxifen with RT may not be detrimental to rates of local control in patients^9,10^ despite tamoxifen-mediated cell cycle arrest in G1, a more radioresistant phase of the cell cycle^11^. Although there is a lack of conclusive evidence available to support concurrent versus adjuvant administration of tamoxifen with RT, multiple studies have demonstrated an increase in local control with the administration of tamoxifen and radiation therapy (RT) compared to RT alone^12,13^. Ongoing clinical trials, including REaCT-RETT (NCT03948568), CONSET (NCT00896155), and STARS (NCT00887380), are assessing the use of anti-estrogen therapies in combination with radiation to evaluate the toxicity and efficacy of sequential versus concurrent administration of treatment clinically in women with breast cancer.

In addition, the ongoing global COVID-19 pandemic has in many cases necessitated changes to the standard treatment for women with early-stage ER+ breast cancer, with neoadjuvant ET used much more frequently as a bridge to surgery^14–16^. These women, who initiated ET while breast surgeries were delayed, often continued their ET treatment while receiving adjuvant radiation therapy. Whether this change in practice will impact clinical outcomes for women with early-stage breast cancer remains unclear, and whether concurrent ET with RT is helpful or harmful is again of significant clinical interest. We sought to determine whether endocrine therapy administered concurrently with radiotherapy had a radiosensitizing or radioprotective effect and evaluated whether SERMs, SERDs, estrogen-depleted, or estradiol-stimulated conditions had differential effects on radiosensitization using multiple ER + breast cancer cell lines and an in vivo xenograft model. Having observed radiosensitization with ER inhibition, we also sought to understand the mechanism of estrogen-mediated DNA damage repair in response to RT.

## Results

### Short-term ER inhibition or degradation radiosensitizes ER+ breast cancer cells

**Fig. 1:**
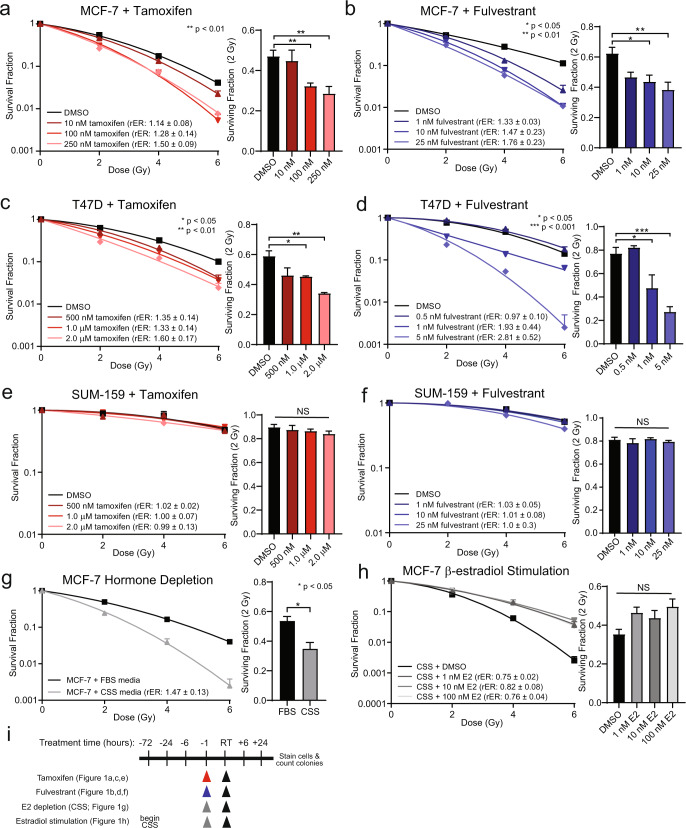
Radiosensitization of ER+ breast cancer cell lines with anti-estrogen therapies. Clonogenic survival assays indicate concentration-dependent radiosensitization of ER+ MCF-7 cells with tamoxifen (**a**) or fulvestrant (**b**). Radiosensitization was also observed in T47D cells with tamoxifen (**c**) or fulvestrant (**d**) treatment, but not in the ER-negative SUM-159 cells treated with tamoxifen (**e**) or fulvestrant (**f**). Clonogenic survival assays were performed in MCF-7 cells pretreated with CSS for 1 h compared to FBS-treated cells (**g**), or MCF-7 cells pretreated for 3 days with CSS before stimulation with β-estradiol (**h**). Clonogenic treatment times are displayed in a schematic (**i**). Data from three or four replicate experiments are graphed as mean ± SEM. (*P < 0.05; **P < 0.01; ***P < 0.001; NS = not significant).

Anti-estrogen therapies, including SERMs, SERDs, and AIs, are effective single-agent therapies that inhibit the growth of ER+ breast cancer cells reliant on estrogen signaling. The efficacy of combination therapy with ionizing radiation, however, remains unclear. To assess radiosensitization in vitro, clonogenic survival assays were first performed with the SERM, tamoxifen, in breast cancer cell lines. The ER+ breast cancer cell lines, MCF-7 or T47D, or the ER-negative cell line, SUM-159, were treated with sub-IC50 concentrations of tamoxifen for one hour prior to radiation treatment. Radiosensitization was observed with a 1 h pretreatment of tamoxifen in MCF-7 cells with radiation enhancement ratios (rER) of 1.14–1.50 with 10–250 nM tamoxifen (Fig. 1a) and rER of 1.33–1.60 with 500 nM-2.0 μM tamoxifen in T47D cells (Fig. 1c). Interestingly, little radiosensitization was observed in MCF-7 cells when tamoxifen was given 6 h before radiation treatment (rER: 0.99–1.10, Supplementary Fig. 1a) or when tamoxifen was given 24 h before radiation (rER: 0.98–1.09, Supplementary Fig. 1b). In contrast to results in ER+ cell lines, RT-induced cell death of ER-negative SUM-159 cells was not potentiated with the addition of tamoxifen (Fig. 1e). Pretreatment for one hour with tamoxifen did not sensitize SUM-159 cells to RT as there was no observed increase in radiosensitization (rER: 0.99–1.02) or decrease in the surviving fraction of cells at 2 Gy (SF-2Gy) with tamoxifen treatment.

Radiosensitization was also assessed after treatment with the SERD, fulvestrant, given one hour prior to RT. ER+ MCF-7 cells treated with 1–25 nM fulvestrant had rER of 1.33–1.76 (Fig. 1b). Similar levels of radiosensitization were observed in T47D cells with 0.5–5 nM fulvestrant (rER: 0.97–2.81, Fig. 1d), with a statistically significant decrease in the SF-2Gy in both cell lines. In contrast to results with tamoxifen, extended pretreatment with fulvestrant for 6 or 24 h in MCF-7 cells resulted in comparable levels of radiosensitization as observed with 1 h pretreatment (6 h rER: 1.42–1.49, Supplementary Fig. 1c; 24 h rER: 1.51–1.89, Supplementary Fig. 1d). These data suggest that the delayed administration of radiation following treatment with tamoxifen, but not fulvestrant, may be less effective than the shorter timeline of treatment in vitro. These observed differences may be explained by the kinetics of degradation of ERα protein in MCF-7 and T47D cells in which maximal degradation occurs 4–6 h post-fulvestrant treatment (Supplementary Fig. 1e, f). Treatment with fulvestrant also did not radiosensitize ER-negative SUM-159 cells (rER: 1.0–1.03, Fig. 1f).

Next, we used the investigational oral SERD, AZD9496, to assess radiosensitization in ER+ breast cancer cell lines in vitro. ER+ MCF-7 cells were treated with 100–500 nM AZD9496 for 1 h prior to RT, and radiosensitization was observed (rER: 1.36–1.56, Supplementary Fig. 2c). T47D cells were more sensitive to AZD9496 treatment, and radiosensitization was achieved with 100 pM-1.0 nM (rER: 1.00–1.33, Supplementary Fig. 2d). SUM-159 cells, a triple-negative breast cancer (TNBC) cell line lacking ER expression, had no change in sensitivity to radiation with AZD9496 treatment (rER: 1.06–1.07, Supplementary Fig. 2e). Together, these results suggest that pharmacologic inhibition (tamoxifen) or degradation (fulvestrant, AZD9496) of ER is sufficient to radiosensitize ER+ breast cancer cells but not ER-negative breast cancer cells.

We also wanted to explore whether the sequence of treatment mattered as recent data have suggested that this may be important for the activity of immune agents for breast cancer cell deaths^17^. Clonogenic survival assays were performed in which fulvestrant treatment was administered 6 or 24 h after RT to see if there were effects on radiosensitization or cellular survival. Treatment of MCF-7 cells first with RT, followed 6 h later with fulvestrant, resulted in similar levels of radiosensitization as observed when fulvestrant was given prior to radiotherapy (rER: 1.23–1.49, Supplementary Fig. 1g). Treatment of MCF-7 cells with fulvestrant 24 h after RT resulted in only a slight radiosensitization (rER: 1.03–1.25, Supplementary Fig. 1h), suggesting that delayed administration of fulvestrant after RT (24 h) is not sufficient to promote the radiosensitization phenotype. Together these findings indicate that fulvestrant treatment, given prior to or shortly after radiotherapy (6 h), is sufficient to promote radiosensitization of ER+ MCF-7 cells in vitro.

To further investigate the role of estrogen in promoting radioresistance, we performed clonogenic survival assays with cells treated with growth medium containing fetal bovine serum (FBS) compared to cells that were pretreated with charcoal-stripped bovine serum (CSS) to remove hormones and growth factors. These conditions mimic the use of aromatase inhibitors which are used to lower levels of estrogens. When MCF-7 cells were pretreated with CSS for one hour prior to RT, radiosensitization was observed relative to FBS-treated MCF-7 cells (rER: 1.47 ± 0.13, Fig. 1g). Similarly, T47D cells pretreated with CSS for one hour prior to RT were radiosensitized relative to FBS-treated T47D cells (rER: 1.51 ± 0.10, Supplementary Fig. 2a). Stimulation with estradiol for one hour prior to RT was also sufficient to provide a radioprotective effect in hormone-stripped MCF-7 cells (rER: 0.75–0.82, Fig. 1h) and hormone-stripped T47D cells (rER: 0.51–0.57, Supplementary Fig. 2b). Therefore, restriction of estrogens in these experiments was sufficient to promote radioresistance, where stimulation with estradiol re-established the radioresistant phenotype in ER+ MCF-7 and T47D cells pretreated with CSS in vitro. Schematics outlining the various treatment conditions for clonogenic survival assays are shown (Fig. 1i, Supplementary Fig. 1i, and Supplementary Fig. 2f). Together these results suggest a role for estrogens in promoting radioresistance in ER+ breast cancer models.

### ER-targeting therapies inhibit dsDNA break repair through impaired NHEJ efficiency

**Fig. 2:**
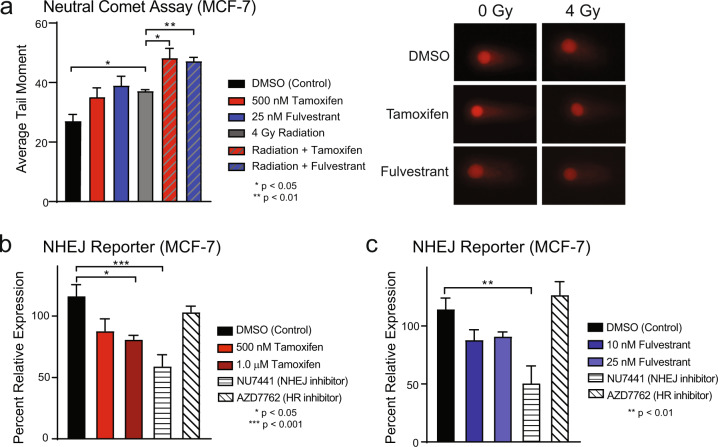
Tamoxifen inhibits double-strand DNA break repair and NHEJ efficiency in MCF-7 cells. The neutral comet assay was used to assess dsDNA break repair in MCF-7 cells treated with ± 500 nΜ tamoxifen ± 25 nM fulvestrant ± 4 Gy RT (**a**). Representative images of comets are shown. NHEJ efficiency in MCF-7 cells was assessed using a transient pEYFP reporter construct. Cells were treated with tamoxifen (**b**) or fulvestrant (**c**) with AZD7762, a Chk1/2 inhibitor, used as a negative control and NU7441, a DNAPK inhibitor, used as a positive control. Data from triplicate experiments are graphed as mean ± SEM. (*P < 0.05; **P < 0.01; ***P < 0.001).

Ionizing radiation induces both single- and double-strand DNA (dsDNA) breaks, though double-strand breaks are more difficult to repair and are more likely to be lethal^18^. One mechanism of radiosensitization is through altered DNA damage repair in which decreased efficiency of dsDNA break repair results in increased cell death. To assess the repair of potentially lethal dsDNA breaks and distinguish dsDNA breaks from the more readily repaired single-strand DNA breaks, the neutral comet assay was performed in MCF-7 cells. Cells were treated with vehicle control (DMSO), 500 nM tamoxifen, or 25 nM fulvestrant for one hour prior to RT, then harvested 6 h after treatment with 4 Gy RT and residual unrepaired dsDNA breaks were measured. The average tail moment was recorded with longer tail moments corresponding to a higher number of unresolved dsDNA breaks. As expected, treatment with radiation was sufficient to induce more dsDNA breaks compared to control, with significantly longer tail moments in the RT group. Combination treatment of tamoxifen with RT or fulvestrant with RT resulted in an increase in dsDNA breaks compared to RT alone (average tail moment in control: 27.1, tamoxifen: 35.1, fulvestrant: 39.0, RT: 37.1, RT + tamoxifen: 48.2, RT + fulvestrant: 47.2, Fig. 2a). Representative images of comet tails are shown (Fig. 2a). These results suggest that endocrine therapy treatment leads to increased dsDNA breaks at this timepoint when ET is given concurrently with RT.

DNA double-strand breaks are primarily repaired through the NHEJ pathway or through homologous recombination (HR). To determine whether these dsDNA break repair mechanisms were altered with ER-targeting therapies, the efficiency of NHEJ and HR was assessed using multiple, non-overlapping assays for NHEJ and HR. A pEYFP reporter system was first used to measure NHEJ efficiency. Cells were treated with tamoxifen or fulvestrant as well as controls including NU7441, an inhibitor of DNA-dependent protein kinase (DNAPK), an important part of the NHEJ pathway, or AZD7762, a pharmacologic inhibitor of Chk1/2, which are essential proteins for an efficient HR response. In MCF-7 cells, treatment with 1.0 μM tamoxifen decreased NHEJ efficiency 30% compared to control (Fig. 2b). Treatment with 10–25 nM fulvestrant also had a relative decrease in NHEJ efficiency (10 nM: 24%, 25 nM: 20%, Fig. 2c). Taken together with the results from the comet assay, where RT with tamoxifen or fulvestrant led to an increase in unresolved dsDNA breaks, these findings suggest that the unresolved DNA breaks are a result, at least in part, of inhibited NHEJ activity after treatment with tamoxifen or fulvestrant.

**Fig. 3:**
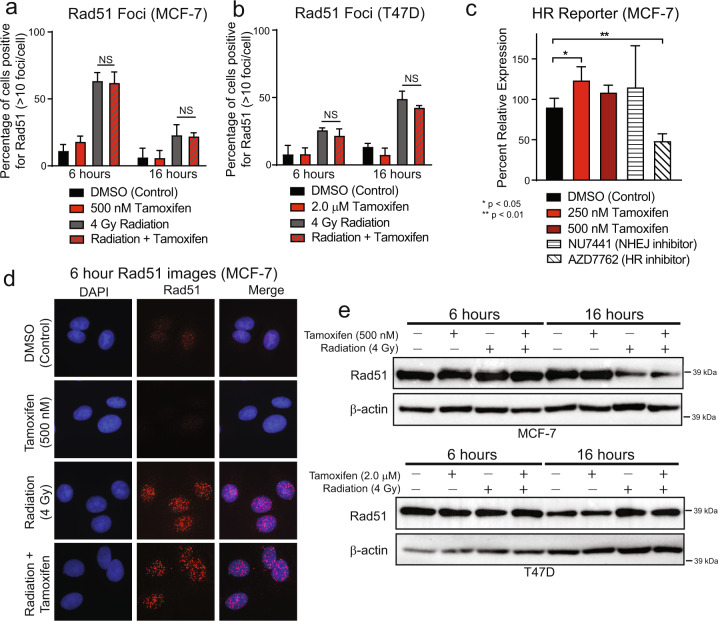
Tamoxifen does not inhibit homologous recombination efficiency. Immunofluorescence was used to stain Rad51 foci in MCF-7 cells treated with ±500 nM tamoxifen ± 4 Gy RT (**a**) and in T47D cells treated ± 2.0 μM tamoxifen ± 4 Gy RT (**b**). A stable homologous recombination reporter construct was used to assess HR efficiency in MCF-7 cells treated with tamoxifen (**c**). AZD7762, a Chk1/2 inhibitor, was used as a positive control; NU7441, a DNAPK inhibitor, was used as a negative control. Representative images of MCF-7 Rad51 foci at the 6 h timepoint are shown (**d**). Total Rad51 protein levels were assessed by western blot in MCF-7 and T47D cells treated ± tamoxifen ± RT at 6- and 16-h post-RT (**e**). Data from three or four replicate experiments are graphed as mean ± SD. (*P < 0.05; **P < 0.01; NS = not significant).

HR was assessed by observing Rad51 foci as a marker for active repair. In MCF-7 cells, treatment with tamoxifen and radiation induced an increase in Rad51-positive cells at 6 h post-RT (Fig. 3a). There was no statistically significant difference in the percentage of Rad51-positive cells when comparing those treated with RT alone compared to the combination of tamoxifen with RT at 6 h. This effect persisted with no change in Rad51-positive cells in RT versus combination-treated cells even as dsDNA breaks were repaired at 16 h post-RT. Similar results were observed in T47D cells (Fig. 3b), and representative images are shown (Fig. 3d and Supplementary Fig. 3). In addition, no changes were observed in total Rad51 protein expression as observed by western blot at 6- or 16-h post-RT (Fig. 3e).

To further confirm these findings, a stable HR reporter system was used to assess HR efficiency in MCF-7 cells. Cells treated with the positive control, AZD7762, had, as expected, a statistically significant decrease (46%) in HR efficiency. Cells treated with tamoxifen or the negative control, NU7441, had no change in HR efficiency or a slight increase in HR efficiency, respectively (250 nM tamoxifen: 37% increase, 500 nM tamoxifen: 20% increase, NU7441: 28% increase, Fig. 3c), suggesting that HR efficiency is not altered by ER inhibition with tamoxifen in MCF-7 cells. Taken together, these data indicate that the efficiency of NHEJ, but not of HR, is negatively affected by anti-estrogen therapy in ER+ models of breast cancer and may be contributing to the radiosensitization phenotype observed with concurrent administration of anti-estrogen therapy and RT.

### Cell-cycle arrest is induced with radiation or short-term endocrine therapy treatment

Endocrine therapies used for ER+ breast cancer treatment are known to cause G1 cell cycle arrest in vitro^19^, but the effect of concurrent endocrine therapy and radiation on the cell cycle has not been established. To determine whether cell cycle arrest was contributing to the observed radiosensitization with tamoxifen or fulvestrant treatment, cell cycle progression was assessed in MCF-7 and T47D cells following treatment. Cells were treated with tamoxifen or fulvestrant for one hour before RT, and cell cycle was assessed at 6-, 16-, and 24-h post-RT. Changes in cell cycle distributions were not observed until 16 h post-RT, when RT-induced G1 arrest was observed in p53 wild-type MCF-7 cells (control: 40.6% G1, RT: 70.9% G1; Supplementary Fig. 4a) and radiation-induced G2 arrest was observed in p53-mutant T47D cells (control: 74.5% G1, RT: 36.5% G1; Supplementary Fig. 4b). Treatment with tamoxifen alone or in combination with radiotherapy did not result in changes to cell cycle distribution at 24 h compared to control cells or cells treated with RT alone, respectively (MCF-7: tamoxifen: 42.5% G1, RT + tamoxifen: 71.0% G1; T47D: tamoxifen: 79.7% G1, RT + tamoxifen: 40.1% G1). Similar results were observed at 16 h post-RT with fulvestrant treatment in MCF-7 (control: 29.9% G1, fulvestrant: 36.9% G1, RT: 62.1% G1, RT + fulvestrant: 63.2%; Supplementary Fig. 4c) or T47D cells (control: 53.7% G1, fulvestrant: 60.1% G1, RT: 20.6% G1, RT + fulvestrant: 23.2% G1; Supplementary Fig. 4d). At 24 h, there was an increase in G1 arrest in T47D cells that were treated with tamoxifen or fulvestrant alone; however, cells treated with the combination of RT with tamoxifen or RT with fulvestrant remained arrested in G1 (MCF-7) or G2 (T47D). Corroborating data were observed by western blot in which minimal changes were observed in the expression of cyclins A, B, and E in T47D cells at 6 h post-RT, but by 16 h post-RT there was a substantial increase in expression of cyclins A and B, suggesting arrest in G2/M (Supplementary Fig. 4f). Expression of cyclins A and B is increased at 24 h but resolved by 48 h post-RT. Increased expression of cyclins A and B was not observed in MCF-7 cells, but rather an increase in cyclin E expression, corresponding to arrest in G1 (Supplementary Fig. 4e), was also observed by western blot. Taken together, these data indicate that treatment with tamoxifen or fulvestrant causes arrest in G1 in both MCF-7 and T47D cells after prolonged treatment. G1 arrest, however, is achieved only after 24 h, and not on a timeline that is relevant to explain the observed radiosensitization. Treatment with RT alone or in combination with tamoxifen or fulvestrant also results in G1 or G2 arrest in p53 wild-type or mutant cells, respectively, further suggesting that when given in combination with RT, ER-targeting therapies do not promote radiosensitization through a cell cycle-mediated mechanism.

### Apoptosis is not induced with endocrine therapies in combination with radiation

Induction of apoptosis is also a potential mechanism for the radiosensitization that was observed with endocrine therapies^20–22^. Treatment with tamoxifen has been shown to induce apoptosis^20^ while estrogens have been shown to induce or inhibit apoptosis in different contexts^21^. Clinically, treatment with tamoxifen or anastrozole does not result in a change in the apoptotic index over time^22^. To understand whether the combination of tamoxifen or fulvestrant with RT was increasing the percentage of cells undergoing apoptosis, and therefore increasing radiosensitivity, apoptosis was assessed by multiple non-overlapping assays including Annexin V/PI-based flow cytometry and cleaved PARP formation by western blotting at 48 h post-RT. In MCF-7 cells, treatment with tamoxifen alone (500 nM), radiation alone (4 Gy), or the combination treatment did not increase the percentage of cells undergoing apoptosis as observed by flow cytometry (control: 10.2% tamoxifen: 11.2%, RT: 10.8%, RT + tamoxifen: 10.2%; Supplementary Fig. 5a). Similarly, in T47D cells, no statistically significant changes in the percentage of cells undergoing apoptosis were observed with treatment of tamoxifen alone (2.0 μM), or RT alone, although there was a slight increase in apoptosis with combination treatment compared to control (control: 4.2%, tamoxifen: 4.9%, RT: 6.7%, RT + tamoxifen: 10.4%; Supplementary Fig. 5b). In addition, there were no changes in cleaved PARP, suggesting that the increase in radiosensitization with tamoxifen is not due to an increase in apoptosis in MCF-7 and T47D cells (Supplementary Fig. 5c, d). Using the flow-based approach, cells treated with fulvestrant or fulvestrant in combination with RT did not induce apoptosis compared to RT alone in MCF-7 (control: 9.4%, fulvestrant: 9.4%, RT: 10.7%, RT + fulvestrant: 15.6%; Supplementary Fig. 5e) or T47D (control: 6.8%, fulvestrant: 11.1%, RT: 15.3%, RT + fulvestrant: 21.2%; Supplementary Fig. 5f) cells. Taken together these experiments indicate that radiosensitization was not due to apoptosis in response to the combination treatment of endocrine therapy with RT.

### Endocrine therapies induce senescence alone and in combination with radiation therapy

**Fig. 4:**
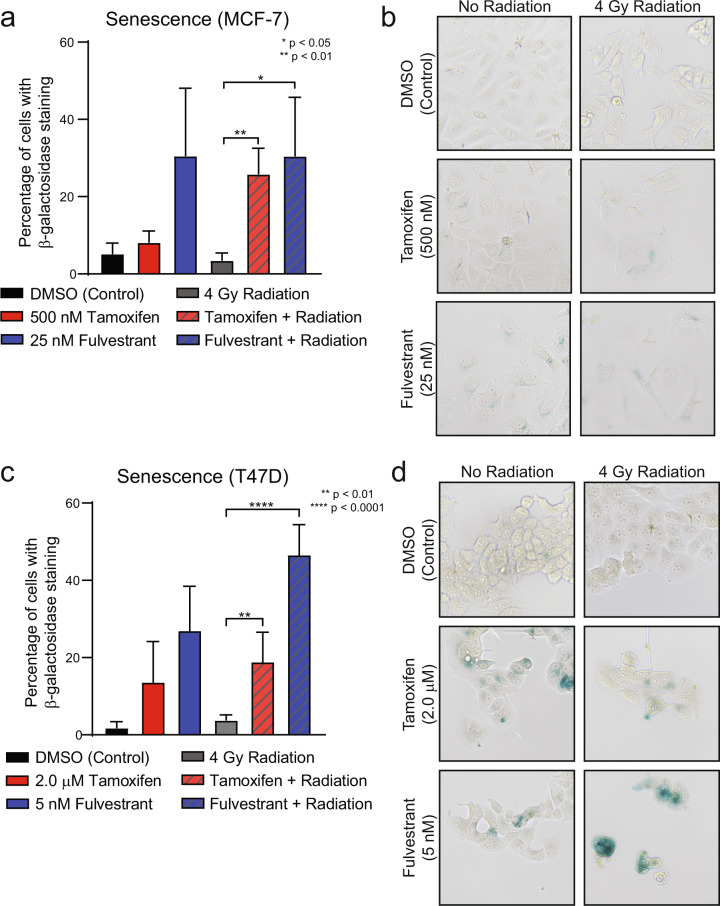
Endocrine therapies in combination with radiation induce senescence. MCF-7 cells were treated with 500 nM tamoxifen or 25 nM fulvestrant for one hour prior to 4 Gy radiation and stained for β-galactosidase at 14 days post-RT. Quantification of cells positive for β-galactosidase was performed for MCF-7 (**a**) and T47D (**c**) cells. Representative images of β-galactosidase staining are shown for each cell line (**b**, **d**). Data from three or four replicate experiments are graphed as mean ± SD. (*P < 0.05; **P < 0.01; ****P < 0.0001).

Estrogens and ERα signaling have also been recognized for their contributions toward inhibiting cellular senescence and promoting cellular growth^23,24^. Furthermore, induction of cellular senescence is a well-described phenomenon of several classes of radiosensitizing drugs^25–27^. Treatment with RT alone has also been shown to induce senescence^28–30^; however, the impact of RT on breast cancer cells specifically is not well-characterized. In our models, β-galactosidase staining was used to determine whether treatment with tamoxifen or fulvestrant alone or in combination with RT may induce an increase in senescence in vitro in breast cancer cell lines. MCF-7 cells treated with tamoxifen or fulvestrant alone had a marked increase in cells positive for β-galactosidase staining (Fig. 4a). In cells treated with the combination of tamoxifen or fulvestrant with RT, however, there was a substantial increase in β-galactosidase-positive cells suggesting that the combination of RT with tamoxifen or RT with fulvestrant resulted in an overall increase in senescence (control: 5.3%, tamoxifen: 8.2%, fulvestrant: 30.6%, RT: 3.6%, RT + tamoxifen: 25.9%, RT + fulvestrant: 30.6%, Fig. 4a). This was confirmed visually with representative images of each treatment (Fig. 4b). Similar results were observed in T47D cells treated with tamoxifen in combination with RT or fulvestrant in combination with RT in which there was an induction of senescence with tamoxifen or fulvestrant treatment alone, but this increase was magnified in cells treated with ET in combination with RT (control: 1.9%, tamoxifen: 13.7%, fulvestrant: 27.1%, RT: 3.9%, RT + tamoxifen: 19.0%, RT + fulvestrant: 46.7%, Fig. 4c, d). These findings suggest that induction of senescence is responsible, at least in part, for the observed radiosensitization with combination ET and RT in preclinical ER+ breast cancer models.

### Tamoxifen is synergistic with radiation in an in vivo xenograft model

**Fig. 5:**
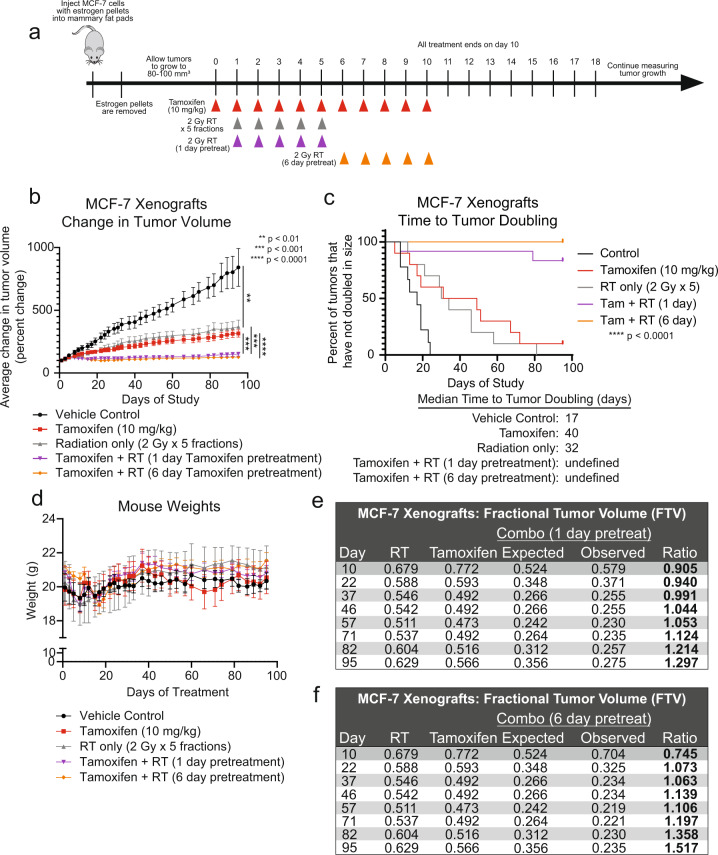
Tamoxifen in combination with radiation is more effective than radiation alone in an MCF-7 xenograft model. MCF-7 cells were injected into the mammary fat pads of CB17-SCID mice, and treatment was initiated when tumors were 80–100 mm^3^. Mice treated with the combination of tamoxifen and radiation received tamoxifen for 1 or 6 days prior to the start of radiotherapy (**a**). The average change in tumor volume was recorded for each treatment condition (**b**). Time to tumor doubling was assessed for each treatment (**c**). The combination treatment did not have significant toxicity as there were no changes in mouse weights (**d**). Tamoxifen with radiation was found to be synergistic using the fractional tumor volume method in which ratios >1 indicate synergy (**e**, **f**). Data are graphed as mean ± SEM. (**P < 0.01, ***P < 0.001, ****P < 0.0001).

Next, to further assess radiosensitization in an in vivo xenograft model, MCF-7 cells were injected subcutaneously into the mammary fat pads of CB17-SCID mice. The resultant tumors were treated with tamoxifen alone (10 mg/kg), RT alone (5 × 2 Gy fractions), or tamoxifen and RT with variable timing of the RT in the combination groups (Fig. 5a). To strictly assess radiosensitization, all tamoxifen treatments were stopped after the first 11 days of treatment. Tamoxifen was given concurrently with the fractions of administered radiation and discontinued so as to eliminate confounding effects as a result of single-agent tamoxifen treatment. When assessing tumor volume, compared to control, there was a significant reduction in the tumor volume for mice treated with tamoxifen alone or RT alone (Fig. 5b). Notably, the mice receiving both tamoxifen and RT had a statistically significant reduction in tumor volume compared to tamoxifen or radiation treatment alone, and the combination treatment resulted in a delay in time to tumor doubling (17 days for control, 40 days for tamoxifen only, 32 days for RT only, undefined for tamoxifen + RT, Fig. 5c). In addition, there were no notable changes in the weights of the mice, suggesting that treatments were well-tolerated (Fig. 5d). Synergy was assessed with the fractional tumor volume (FTV) method^31^, which demonstrated that compared to treatment with RT or tamoxifen alone, the combination of tamoxifen with RT with either a 1-day pretreatment (Fig. 5e) or 6-day pretreatment (Fig. 5f) of tamoxifen was synergistic and not just additive. Together, these in vivo data suggest that combining tamoxifen with RT is more effective than monotherapy alone. In addition, there may not be a significant difference in radiosensitization when comparing the 1-day versus 6-day pretreatment of the MCF-7 xenografts as there was no statistically significant change in tumor volume between mice with different pretreatments of tamoxifen.

## Discussion

Here we demonstrate that abrogation of ER signaling with tamoxifen, fulvestrant, or AZD9496 results in radiosensitization of ER+ breast cancer cell lines (Fig. 1 and Supplementary Figs. 1 and 2) through the inhibition of DNA damage repair via NHEJ (Fig. 2) and an induction of cellular senescence with combination treatment of ET and RT (Fig. 4). Changes in HR-mediated repair, apoptosis, and cell cycle were not observed in cells treated with ET and RT compared to cells treated with RT alone (Fig. 3 and Supplementary Figs. 3–5). Estradiol stimulation resulted in radioprotection in ER+ models, and estrogen depletion was radiosensitizing in these same models (Fig. 1 and Supplementary Fig. 2). Radiosensitization was also observed in vivo with concurrent tamoxifen treatment using an MCF-7 xenograft model (Fig. 5). Together, these results propose that treatment with the combination of ER inhibition and RT may be more effective than ER inhibition or RT alone. These data also suggest an expanded role for ER-targeting therapies as radiosensitization agents for patients with ER+ breast tumors and support the continued use of ET during RT when ET is started as a bridging strategy to surgery.

Our work demonstrates that treatment with tamoxifen, fulvestrant, or AZD9496 may radiosensitize ER+ breast cancer models through induction of senescence and inhibition of NHEJ-mediated repair. These findings are consistent with previous work which has demonstrated a decrease in NHEJ efficiency in human fibroblasts undergoing senescence compared to young or presenescent cells^32^. Because cell cycle redistribution was not observed at early time points following tamoxifen or fulvestrant treatment, these results suggest that changes to cell cycle assortment are not driving the radiosensitization phenotype observed with short, one hour pretreatment times in MCF-7 and T47D cells. Cell cycle arrest does occur on timescales that are relevant for the longer fulvestrant pretreatment times prior to radiation, however, similar levels of radiosensitization still occur despite cell cycle redistribution, further suggesting that the observed radiosensitization is not solely based on cell cycle changes. Together our findings do not support the clinical concern of sequential use of radiation with endocrine therapy due to radioresistance resulting from cell cycle redistribution. Rather, our work suggests that concurrent administration of RT with tamoxifen or fulvestrant results in radiosensitization despite cell cycle arrest with longer drug pretreatment times. Previous work from our lab and others demonstrated that antagonism of hormone receptors, notably the androgen receptor, results in radiosensitization of AR+ TNBC^33–35^ and prostate cancer^36–39^. The observed radiosensitization is due, at least in part, to inhibition of NHEJ through downregulation of p-DNAPK. These findings, along with our work outlined here, suggest a broader role for hormone receptors in the regulation of NHEJ efficiency in hormone receptor-positive cancers following ionizing radiation treatment.

Previous studies have also demonstrated the utility of using ER-targeted therapies in combination with RT in ER+ breast cancer models. This work, performed in both in vitro and in vivo models, has been inconclusive in determining the optimal timing of administration of ET and RT for patients. In one study, rats with mammary tumors induced by 1-methyl-1-nitrosourea (MNU) benefited from the treatment of tamoxifen or RT alone, while receiving similar levels of benefit from the combination treatment with no significant radiosensitization noted^40^. Other studies using MCF-7 cells grown in spheroids with estrogen supplementation had little change in radiosensitization compared to cells grown in monolayers; however, cells grown in estrogen-free media had a significant decrease in radiosensitivity compared to cells treated with 17β-estradiol^41^. Treatment with the aromatase inhibitor, letrozole, also had radiosensitizing effects on MCF-7 cells in vitro^42^. In a contrasting study, a decrease in radiosensitivity was observed by Wazer et al. when treating MCF-7 cells with tamoxifen in combination with RT compared to treatment with RT alone when tamoxifen was given 48 h prior to RT^43^. These findings are consistent with our results suggesting that prolonged tamoxifen pretreatment may no longer be sufficient to radiosensitize MCF-7 cells in vitro compared to the radiosensitization we observed with shorter pretreatment times. Treatment of MCF-7 cells with fulvestrant also resulted in significant radiosensitization through a decrease in Rad51 and DNAPKcs protein levels with sustained fulvestrant treatment^44^. While our results suggest that tamoxifen or fulvestrant treatment decreases the efficiency of NHEJ, no changes in Rad51 protein levels were observed (Fig. 3e). Differences in fulvestrant pretreatment times (4 days versus 1 h), however, could explain differences in these results. Together our findings suggest that both SERMs and SERDs can function as radiosensitization agents in p53 wild-type and p53-mutant models of ER + breast cancer and add to a body of literature suggesting this may be an effective clinical strategy, especially in women at high risk for locoregional disease recurrence.

Our current study uses multiple ER+ breast cancer models and suggests that treatment with tamoxifen may sensitize cell and xenograft models to RT in vitro through the inhibition of dsDNA repair in an NHEJ-dependent manner as well as an increase in the induction of senescence with RT in combination with ET. However, there remain limitations to these studies. There are a limited number of model systems used in this work due to the finite number of ER+ breast cancer models that grow ex vivo in culture. In addition, this work uses in vitro or immunocompromised models and therefore cannot address a potential role for the immune system in modulating the radiation response. Extensions of future work could include a more robust investigation of the impact of the immune system using immunocompetent models. Future work will also expand this study using xenograft models with additional ER-targeting agents including additional oral SERDs, an ERα PROTAC degrader, and aromatase inhibitors that require estrogen-depleted media and/or exogenous stable expression of aromatase in the cultured cells. These models will assess radiosensitization in an increasingly diverse set of breast cancer model systems using multiple ER-targeting therapies. This work will also investigate the transcriptional role of ER in the response to radiation to understand how ER may be promoting the transcriptional regulation of genes important for the DNA damage response through canonical ER transcription factor activity.

Importantly, previous clinical studies also raise concerns about the concurrent administration of tamoxifen with RT in regard to the toxicity of treatment. While none of our experiments address this question directly, in our in vivo studies, we saw no evidence of increased toxicity in animals treated with tamoxifen and RT together compared to those treated with RT alone (weight loss, hair loss, dermatitis). In addition, although previous work has demonstrated that tamoxifen is more toxic to the skin when administered in combination with RT^45^, other groups have shown in long-term follow-up studies there are no differences in cosmesis with tamoxifen and RT^46^. Concurrent treatment of tamoxifen with RT has also been shown to result in increased levels of lung fibrosis compared to RT alone^47^. Results from the CO-HO-RT trial (NCT00208273), however, demonstrated that concurrent administration of the aromatase inhibitor, letrozole, with RT provides radiosensitization without an increase in skin toxicity^42^. Future clinical studies can address to what extent, if any, concurrent anti-estrogen therapies contribute to added normal tissue toxicity during and after radiation.

Together our data suggest that the administration of tamoxifen or fulvestrant or depletion of estrogens concurrently with RT may increase the effectiveness of radiotherapy, demonstrating that this could be an effective treatment intensification strategy for patients with locally advanced ER+ breast cancer at high risk for locoregional recurrence. This intensification of therapy could be considered in appropriately selected patients but should be balanced against the ongoing efforts for treatment de-escalation for women with early-stage ER+ breast cancer who may appropriately choose to omit radiation therapy given their low risk of local recurrence^48^. Previous studies have demonstrated that concurrent treatment of tamoxifen with RT does not appear to have an adverse effect on local/systemic control compared to sequential treatment^10^. In addition, the REaCT-RETT trial underway is comparing sequential to concurrent administration of RT with ET and will provide further insight into this clinical question. Future studies will continue to investigate these hypotheses through clinical trials seeking to improve local control and outcomes for patients with ER+ breast cancers. This work remains of clinical importance for the treatment of breast cancer patients especially in light of the COVID-19 pandemic where patients have received endocrine therapy prior to the administration of radiotherapy.

## Methods

### Cell lines

Cells were grown in an incubator at 37 °C with 5% CO_2_. SUM-159 cells received from Dr. Steven P. Ethier (University of Michigan, Ann Arbor, MI) were grown in HAMS F-12 media (ThermoFisher 11765054), supplemented with 6 μg/mL insulin (Sigma I9278), 0.01 M HEPES (Sigma H3375), 1 μg/mL hydrocortisone (Sigma H4001), 1X antibiotic-antimycotic (anti-anti; ThermoFisher 15240062), and 5% FBS (Atlanta Biologicals S11550H). MCF-7 and T47D cells were received from ATCC and grown in DMEM media (ThermoFisher 11965092) containing 1% penicillin/streptomycin (ThermoFisher 15070063) and 10% FBS. Media containing charcoal-stripped bovine serum (Atlanta Biologicals S11650H) was used for indicated assays. For experiments with β-estradiol stimulation, cells were stripped of hormones with media containing CSS for three days prior to stimulation. All cell lines were authenticated by DNA fingerprinting using short tandem repeat (STR) profiling. Cells were routinely tested for mycoplasma using the MycoAlert Mycoplasma Detection kit (Lonza LT07).

### Clonogenic survival assay

Cells were suspended in single cell suspension before plating in six-well plates. Cells were allowed to adhere overnight before treatment with drug-containing media. Drug treatment was given 1–24 h prior to or following radiation (0–6 Gy). Colony growth was allowed for 1–2 weeks, then cells were fixed with methanol/acetic acid and stained with crystal violet. Colonies containing ≥50 cells were counted and analyzed using the linear-quadratic method. Experiments were repeated in triplicate, and SF-2Gy values are represented as the mean ± SEM.

### Neutral comet assay

Cells were plated in six-well plates and allowed to adhere overnight. The following morning, cells were treated with drug media for one hour before radiation treatment (4 Gy). At 6 h after radiation, cells were harvested with trypsin, suspended in low melting point agarose (ThermoFisher 15-455-200), and pipetted onto a CometSlide (Trevigen 4250-050-03). When agarose had adhered to the slide, the slides were lysed overnight at 4 °C using Comet Assay Lysis Solution (Trevigen 4250-050-01). Following lysis, slides were immersed in TBE buffer containing 90 mM tris buffer, 90 mM boric acid, and 2 mM Na_2_EDTA (pH 8.0). Cells were separated using electrophoresis, then washed, and neutralized in distilled water. Once dry, cells were stained using propidium iodide to stain for DNA. Images were then taken off at least 50 comets per treatment condition using a Nikon Fluorescent Microscope and analyzed using Comet Assay IV (software version 4.3). Results were pooled for statistical analyses, and data are shown as the mean ± SEM for triplicate experiments.

### NHEJ reporter assay

A pEYFP plasmid (gift from Canman lab at the University of Michigan, Ann Arbor, MI) was linearized and purified as previously described^49^. MCF-7 cells were plated in six-well plates, and the following morning treated with tamoxifen, NU7441, or AZD7762 for one hour before transfection. Following pretreatment, cells in each well were transfected with 1 μg of linearized pEYFP plasmid using Lipofectamine 2000 (Invitrogen 11668) and OptiMEM media (Invitrogen 31985-062). Cells were harvested with trypsin 6 h after transfection, and plasmid DNA was isolated using the QIAprep Spin Miniprep Kit (Qiagen 27106). Sybr Green (ThermoFisher 4385612) was used to perform real-time quantitative PCR (ΔΔCt) on a QuantStudio6 Flex Real-Time 384-well qPCR system. NHEJ efficiency was assessed with primers to detect GFP expression (Rejoined DNA: F: 5′-GCTGGTTTAGTGAACCGTCAG-3′, R: 5′-GCTGAACTTGTGGCCGTTTA-3′) relative to a plasmid internal control (Uncut DNA: F: 5′-TACATCAATGGGCGTGGATA-3′, R: 5′-AAGTCCCGTTGATTTTGGTG-3′). Relative efficiency was calculated by normalizing the Ct value of the internal control relative to a no-treatment control sample.

### Immunofluorescence

Coverslips were sterilized in ethanol, and cells were plated on coverslips in 12-well plates. Cells were allowed to adhere overnight before treatment with drug-containing media for one hour prior to RT (4 Gy). Following RT, cells were grown until designated time points and fixed using 4% paraformaldehyde (Thermo Scientific J19943K2). Cells were blocked in a solution containing goat serum (ThermoFisher 16210064) and stained using an anti-Rad51 antibody (GeneTex GTX70230, 1:300) and a fluorescent goat anti-mouse secondary antibody (Invitrogen A11005, 1:2000). Nuclei were stained with ProLong Gold antifade reagent with DAPI (Invitrogen P36931). Pictures of >100 cells were taken using a Nikon Fluorescent Microscope. Cells with ≥10 Rad51 foci/cell were counted as positive. Results were pooled for statistical analyses, and data are shown as the mean ± SD for three or four replicate experiments.

### HR reporter assay

As previously described^49^, an HR reporter DR-GFP plasmid was transfected into MCF-7 cells using Lipofectamine 2000 (Invitrogen 11668) according to the manufacturer’s instructions. Cells containing the plasmid were selected with Geneticin (ThermoFisher 10131035) and validated by flow-cytometry for GFP expression. Validated clones were plated in six-well plates and treated with tamoxifen, NU7441, or AZD7762 for one hour before the addition of SceI adenovirus to induce dsDNA breaks^50^. After 48 h, cells were harvested and fixed before analysis by flow cytometry for GFP^+^ cells at the University of Michigan Flow Cytometry Core. Relative expression was calculated in comparison to untreated control cells for each experiment.

### Western blotting

Cells were plated, allowed to adhere overnight, and pretreated with tamoxifen or fulvestrant one hour prior to irradiation. Cells were harvested at indicated time points and lysed using RIPA buffer (ThermoFisher 89901) containing protease and phosphatase inhibitors (Sigma-Aldrich PHOSS-RO, CO-RO; Santa Cruz Biotechnology sc-3540, sc-24988A; Cayman Chemical 14333, 14405). Samples were separated on precast NuPAGE Bis-Tris protein gels (ThermoFisher), transferred to PDVF membranes (Millipore IPVH00010), and blocked with blotting grade blocker (BioRad 1706404, 5% milk). Antibodies used for protein detection include ERα (Cell Signaling 8644S; 1:1000), Rad51 (Millipore PC130; 1:500), β-actin (Cell Signaling 12262S; 1:50,000), total PARP1 (Abcam ab6079; 1:1000), cleaved PARP (Cell Signaling 5625S; 1:1000), cyclin E1 (Santa Cruz sc-247; 1:1000), cyclin B (Santa Cruz sc-245; 1:1000), and cyclin A (Santa Cruz sc-271682; 1:1000). Secondary antibodies used include anti-mouse (Cell Signaling 7076S; 1:10,000) and anti-rabbit (Cell Signaling 7074S; 1:10,000). All blots are derived from single experiments and are processed in parallel. Quantification of western blots was performed with ImageJ software.

### Flow cytometry

Cells were plated and treated with a drug for one hour before radiation treatment. Following radiation (4 Gy), cells were harvested at predetermined time points. For cell cycle experiments, cells were fixed in 70% ethanol at 6-, 16-, and 24-h post-RT. Before analysis, fixed cells were resuspended in 1× PBS and stained with propidium iodide and RNase (Qiagen 19101). For analysis of apoptosis, cells were harvested by trypsinization 48 h after radiation and stained with annexin V and propidium iodide (Roche 11858777001) immediately preceding analysis. Both apoptosis and cell cycle samples were analyzed at the University of Michigan Flow Cytometry Core using the BioRad ZE5 Cell Analyzer (Supplementary Figs. 6 and 7). Data are shown as mean ± SD for triplicate experiments.

### Drug information

Tamoxifen was obtained from MedChemExpress (HY-13757A). Fulvestrant was obtained from MedChemExpress (HY-13636). AZD9496 was obtained from MedChemExpress (HY-12870). NU7441, an inhibitor of DNA protein kinase catalytic subunit (DNAPKcs), was obtained from Selleckchem (Ku-57788). AZD7762, an inhibitor of Chk1/2, was purchased from Sigma (SML0350). β-estradiol was obtained from MedChemExpress (HY-B0141). All compounds were solubilized in DMSO.

### Animal experiments

MCF-7 cells suspended in 50% Matrigel (ThermoFisher CB-40234) were injected into bilateral mammary fat pads of CB17-SCID mice (6 × 10^6^ cells/injection). Simultaneously on the day of injection, estrogen pellets (Innovative Research of America SE-121) were subcutaneously implanted at the nape of the neck. Pellets were removed when tumors were palpable. Mice were randomized into five groups when tumors had reached 80–100 mm^3^ with 9–12 tumors/group. Mice were then treated with one of the following treatment options (Fig. 5a): vehicle (corn oil: Sigma C8267), tamoxifen only for 11 days, radiation only, or a combination of tamoxifen with radiation. Treatment of tamoxifen was administered by oral gavage for 11 days at a dose of 10 mg/kg. Radiation treatment was administered in five fractions of 2 Gy. Mice in the first combination group received tamoxifen one day before starting radiation therapy, then received concurrent tamoxifen with radiation for five days, followed by five days of tamoxifen therapy following the completion of radiation. Mice in the second combination group received six days of tamoxifen prior to beginning radiation therapy and received five doses of radiation with concurrent tamoxifen (Fig. 5a). Tumor growth was measured 2–3 times/week, and tumor volume was calculated using the equation V = L*W^2^*π/6. Animal protocols and procedures are approved by the Institutional Animal Care and Use Committee at the University of Michigan (Ann Arbor, MI).

### Irradiation

X-ray irradiation was performed at the University of Michigan Experimental Irradiation Core using a Kimtron IC-225. The dose rate of 2 Gy/min was used in keeping with the previous studies^49,51,52^. For in vitro experiments, a 0.1 mm Cu filter was used, and for in vivo experiments, the filter was 0.4 mm Sn + 0.25 mm Cu.

### Statistical analyses

Statistical analyses were performed in GraphPad Prism 8. For in vitro experiments, a one-way ANOVA was with Dunnett’s test for multiple comparisons was used to compare SF-2Gy values, data from the NHEJ reporter, cell cycle analysis for each timepoint, and apoptosis with annexin V/PI. A two-sided Student’s *t* test was used to compare tail moments for the comet assay, HR reporter, Rad51 foci, and senescence data. For animal experiments, a log-rank (Mantel–Cox) test was performed to compare survival curves. *P* values equal to or less than 0.05 were considered significant. Synergy calculations were performed using fractional tumor volume (FTV) in keeping with previous studies^49,51^.

### Reporting summary

Further information on research design is available in the Nature Research Reporting Summary linked to this article.

## Supplementary information

Reporting Summary Checklist

Resupplied SI file requested by ME

## Data Availability

Data and materials generated during the current study are available from the corresponding author upon reasonable request. No code was generated from these experiments.
