# Publisher Correction: Estrogen receptor inhibition mediates radiosensitization of ER-positive breast cancer models

**DOI:** 10.1038/s41523-022-00418-w

**Published:** 2022-04-20

**Authors:** Anna R. Michmerhuizen, Lynn M. Lerner, Andrea M. Pesch, Connor Ward, Rachel Schwartz, Kari Wilder-Romans, Meilan Liu, Charles Nino, Kassidy Jungles, Ruth Azaria, Alexa Jelley, Nicole Zambrana Garcia, Alexis Harold, Amanda Zhang, Bryan Wharram, Daniel F. Hayes, James M. Rae, Lori J. Pierce, Corey W. Speers

**Affiliations:** 1 Department of Radiation Oncology, University of Michigan, Ann Arbor, MI USA; 2 Rogel Cancer Center, University of Michigan, Ann Arbor, MI USA; 3 Program in Cellular and Molecular Biology, University of Michigan, Ann Arbor, MI USA; 4 Department of Pharmacology, University of Michigan, Ann Arbor, MI USA; 5 Department of Internal Medicine, University of Michigan, Ann Arbor, MI USA

**Keywords:** Targeted therapies, Breast cancer, Non-homologous-end joining, Radiotherapy, Breast cancer

Correction to: *npj Breast Cancer* 10.1038/s41523-022-00397-y, published online 10 March 2022

Supplementary Figs. 1–7 was missing from this article and has now been uploaded. The original article has been corrected.

## Supplementary Information

Supplementary Figures 1-7

