# Supplementary material for: Estrogen receptor inhibition mediates radiosensitization of ER-positive breast cancer models
Source: NPJ Breast Cancer. 2022 Mar 10;8:31. doi: 10.1038/s41523-022-00397-y (PMC8913671; doi:10.1038/s41523-022-00397-y)
Supplement: Supplementary file 1 — Reporting Summary Checklist [file 41523_2022_397_MOESM1_ESM.pdf]

nature portfolio

Corresponding author(s): Corey Speers

Last updated by author(s): Jan 14, 2022

Reporting Summary

Nature Portfolio wishes to improve the reproducibility of the work that we publish. This form provides structure for consistency and transparency in reporting. For further information on Nature Portfolio policies, see our Editorial Policies and the Editorial Policy Checklist.

Statistics

For all statistical analyses, confirm that the following items are present in the figure legend, table legend, main text, or Methods section.

n/a	Confirmed	
☐	☒	The exact sample size (n) for each experimental group/condition, given as a discrete number and unit of measurement
☐	☒	A statement on whether measurements were taken from distinct samples or whether the same sample was measured repeatedly
☐	☒	The statistical test(s) used AND whether they are one- or two-sided<br>*Only common tests should be described solely by name; describe more complex techniques in the Methods section.*
☐	☒	A description of all covariates tested
☐	☒	A description of any assumptions or corrections, such as tests of normality and adjustment for multiple comparisons
☐	☒	A full description of the statistical parameters including central tendency (e.g. means) or other basic estimates (e.g. regression coefficient) AND variation (e.g. standard deviation) or associated estimates of uncertainty (e.g. confidence intervals)
☐	☒	For null hypothesis testing, the test statistic (e.g. F, t, r) with confidence intervals, effect sizes, degrees of freedom and P value noted<br>*Give P values as exact values whenever suitable.*
☒	☐	For Bayesian analysis, information on the choice of priors and Markov chain Monte Carlo settings
☒	☐	For hierarchical and complex designs, identification of the appropriate level for tests and full reporting of outcomes
☒	☐	Estimates of effect sizes (e.g. Cohen's d, Pearson's r), indicating how they were calculated

Our web collection on statistics for biologists contains articles on many of the points above.

Software and code

Policy information about availability of computer code

Data collection	No software was used.
Data analysis	No software was used.

For manuscripts utilizing custom algorithms or software that are central to the research but not yet described in published literature, software must be made available to editors and reviewers. We strongly encourage code deposition in a community repository (e.g. GitHub). See the Nature Portfolio guidelines for submitting code & software for further information.

Data

Policy information about availability of data

All manuscripts must include a data availability statement. This statement should provide the following information, where applicable:

- Accession codes, unique identifiers, or web links for publicly available datasets
- A description of any restrictions on data availability
- For clinical datasets or third party data, please ensure that the statement adheres to our policy

Data and materials generated during the current study are available from the corresponding author upon reasonable request.

March 2021

Field-specific reporting

Please select the one below that is the best fit for your research. If you are not sure, read the appropriate sections before making your selection.

☒ Life sciences ☐ Behavioural & social sciences ☐ Ecological, evolutionary & environmental sciences

For a reference copy of the document with all sections, see nature.com/documents/nr-reporting-summary-flat.pdf

Life sciences study design

All studies must disclose on these points even when the disclosure is negative.

Sample size	All in vitro experiments were performed in 2-4 replicates, with most experiments performed in triplicate. For animal experiments, 9-12 tumors were used for each treatment group to provide sufficient sample size for statistical analyses.
Data exclusions	No data were excluded from analyses.
Replication	All experiments were completed in duplicate or triplicate as indicated in the text and/or figure legends.
Randomization	For animal experiments, mice were randomly allocated to treatment groups.
Blinding	Blinding was used for analysis of Rad51-positive cells for immunofluorescent experiments. Samples were unblinded when analysis was complete.

Reporting for specific materials, systems and methods

We require information from authors about some types of materials, experimental systems and methods used in many studies. Here, indicate whether each material, system or method listed is relevant to your study. If you are not sure if a list item applies to your research, read the appropriate section before selecting a response.

Materials & experimental systems

n/a	Involved in the study	
☐	☒	Antibodies
☐	☒	Eukaryotic cell lines
☒	☐	Palaeontology and archaeology
☐	☒	Animals and other organisms
☒	☐	Human research participants
☒	☐	Clinical data
☒	☐	Dual use research of concern

Methods

n/a	Involved in the study	
☒	☐	ChIP-seq
☐	☒	Flow cytometry
☒	☐	MRI-based neuroimaging

Antibodies

Antibodies used	Rad51 antibody [14B4]: GeneTex GTX70230; Lot 43574<br>Alexa Fluor 594 goat anti-mouse (H+L): Invitrogen A11005; Lot 2110496<br>Estrogen Receptor alpha (D8H8): Cell Signaling 8644S; Lot 8<br>Rad51 (Ab-1): Millipore PC130; Lot 3282244<br>B-actin (8H10D10) mAb (HRP conjugate): Cell Signaling 12262S; Lot 2<br>total PARP1: Abcam ab6079; Lot GR128656-2<br>cleaved PARP (Asp214) (D64E10) XP (R): Cell Signaling 5625S; Lot 13<br>cyclin E (HE12): Santa Cruz sc-247; Lot C2918<br>cyclin B (GNS1): Santa Cruz sc-245; Lot D1118<br>cyclin A (B-8): Santa Cruz sc-271682; Lot D1818<br>anti-mouse IgG, HRP-linked antibody: Cell Signaling 7076S; Lot 35<br>anti-rabbit IgG, HRP-linked antibody: Cell Signaling 7074S; Lot 29
Validation	All antibodies are commercially available and validated by the supplier.

Eukaryotic cell lines

Policy information about cell lines

Cell line source(s)	MCF-7 and T47D cells were obtained from ATCC. SUM-159 cells were generously provided by Dr. Steven P. Ethier from University of Michigan stocks.

Authentication	Cell lines were authenticated by DNA fingerprinting using short tandem repeat profiling.
Mycoplasma contamination	All cell lines tested negative for mycoplasma contamination.
Commonly misidentified lines (See ICLAC register)	*Name any commonly misidentified cell lines used in the study and provide a rationale for their use.*

Animals and other organisms

Policy information about studies involving animals; ARRIVE guidelines recommended for reporting animal research

Laboratory animals	All animals used were female CB-17 SCID mice between 6-8 weeks old when the study began.
Wild animals	This study did not use wild animals.
Field-collected samples	This study did not use samples collected from the field.
Ethics oversight	Animal protocols and procedures are approved by the Institutional Animal Care and Use Committee at the University of Michigan (Ann Arbor, MI).

Note that full information on the approval of the study protocol must also be provided in the manuscript.

Flow Cytometry

Plots

Confirm that:

☒ The axis labels state the marker and fluorochrome used (e.g. CD4-FITC).

☒ The axis scales are clearly visible. Include numbers along axes only for bottom left plot of group (a 'group' is an analysis of identical markers).

☒ All plots are contour plots with outliers or pseudocolor plots.

☒ A numerical value for number of cells or percentage (with statistics) is provided.

Methodology

Sample preparation	All experiments were performed on MCF-7 and T47D cells in vitro.
Instrument	Bio-Rad Ze5 cell analyzer was used for all data collection.
Software	Analysis was performed using FCS Express Flow Cytometry Analysis software.
Cell population abundance	Experiments were performed for cell cycle (PI staining) or apoptosis (Annexin V/PI staining). For both sets of experiments, at least 10,000 cells were analyzed for each condition.
Gating strategy	For experiments measuring apoptosis in cells in vitro, gating was performed by using unstained control cells as well as cells stained only with annexin V or only with PI. Once gates were set for each cell line, they were not modified for each sample corresponding to an additional experimental condition for all samples prepared for the sample replicate of the experiment.

☒ Tick this box to confirm that a figure exemplifying the gating strategy is provided in the Supplementary Information.
