# Supplementary material for: Estrogen receptor inhibition mediates radiosensitization of ER-positive breast cancer models
Source: NPJ Breast Cancer. 2022 Mar 10;8:31. doi: 10.1038/s41523-022-00397-y (PMC8913671; doi:10.1038/s41523-022-00397-y)
Supplement: Supplementary file 2 — Resupplied SI file requested by ME [file 41523_2022_397_MOESM2_ESM.pdf]

Supplementary Figure 1

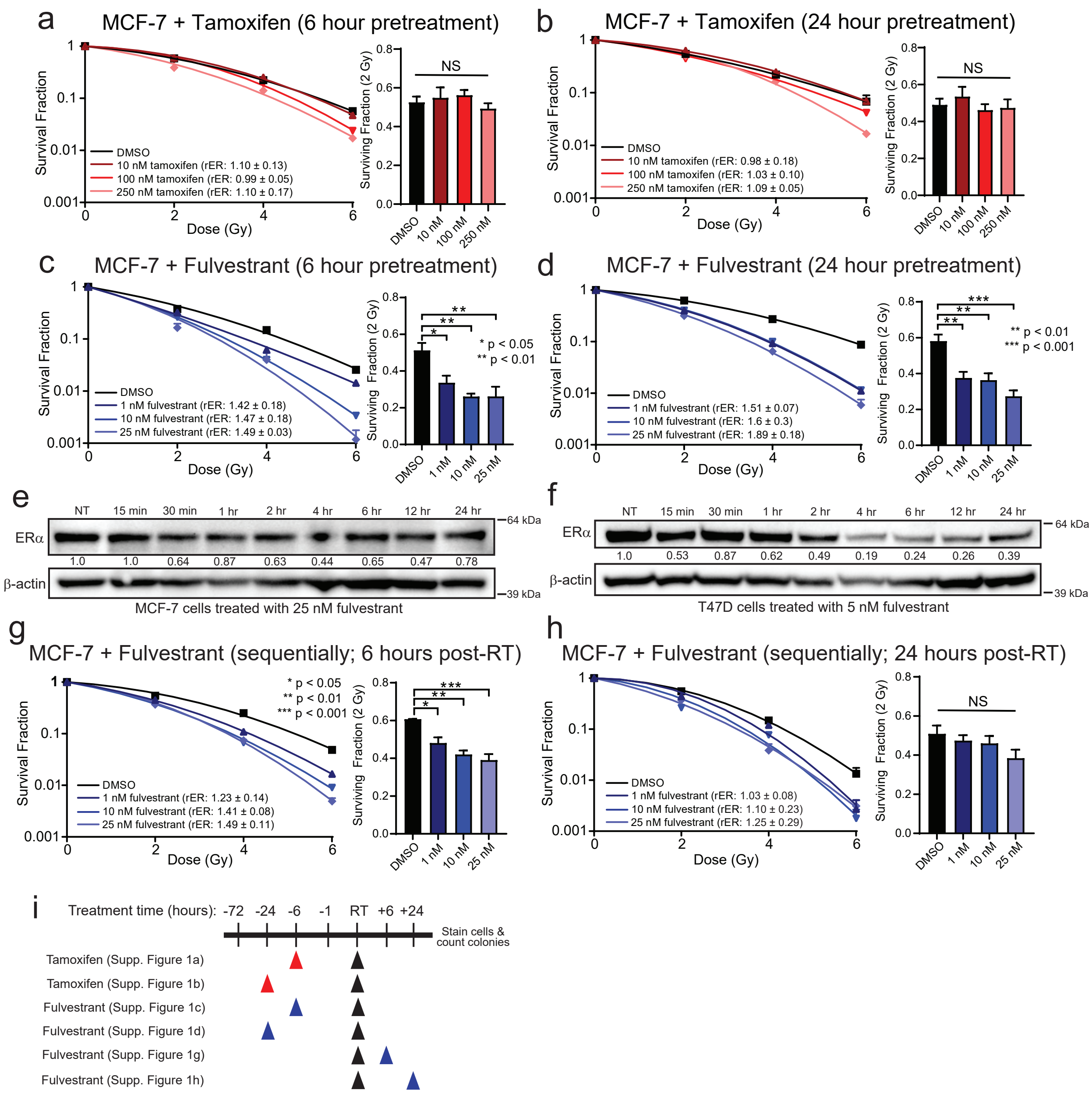

Supplementary Figure 1: Radiosensitization of MCF-7 cells with variable treatment times. Clonogenic survival assays were performed with a 6- (**a**) or 24-hour (**b**) pretreatment with tamoxifen in MCF-7 cells. Similarly, clonogenic survival assays were performed with a 6- (**c**) or 24-hour (**d**) pretreatment with fulvestrant in MCF-7 cells. Western blots assessed the time course of ERα degradation with 25 nM fulvestrant in MCF-7 cells (**e**) or with 5 nM fulvestrant in T47D cells (**f**). Clonogenic survival assays were performed in MCF-7 cells with fulvestrant treatment administered 6 hours (**g**) or 24 hours post-RT (**h**). A schematic of pretreatment times for clonogenic survival assays is shown (**i**). Quantification of western blots is representative of two replicates; quantification is ERα expression relative to no treatment (DMSO) control. Clonogenic data is from triplicate experiments and is graphed as mean ± SEM. (* $p < 0.05$; ** $p < 0.01$; *** $p < 0.001$; NS = not significant)

Supplementary Figure 2

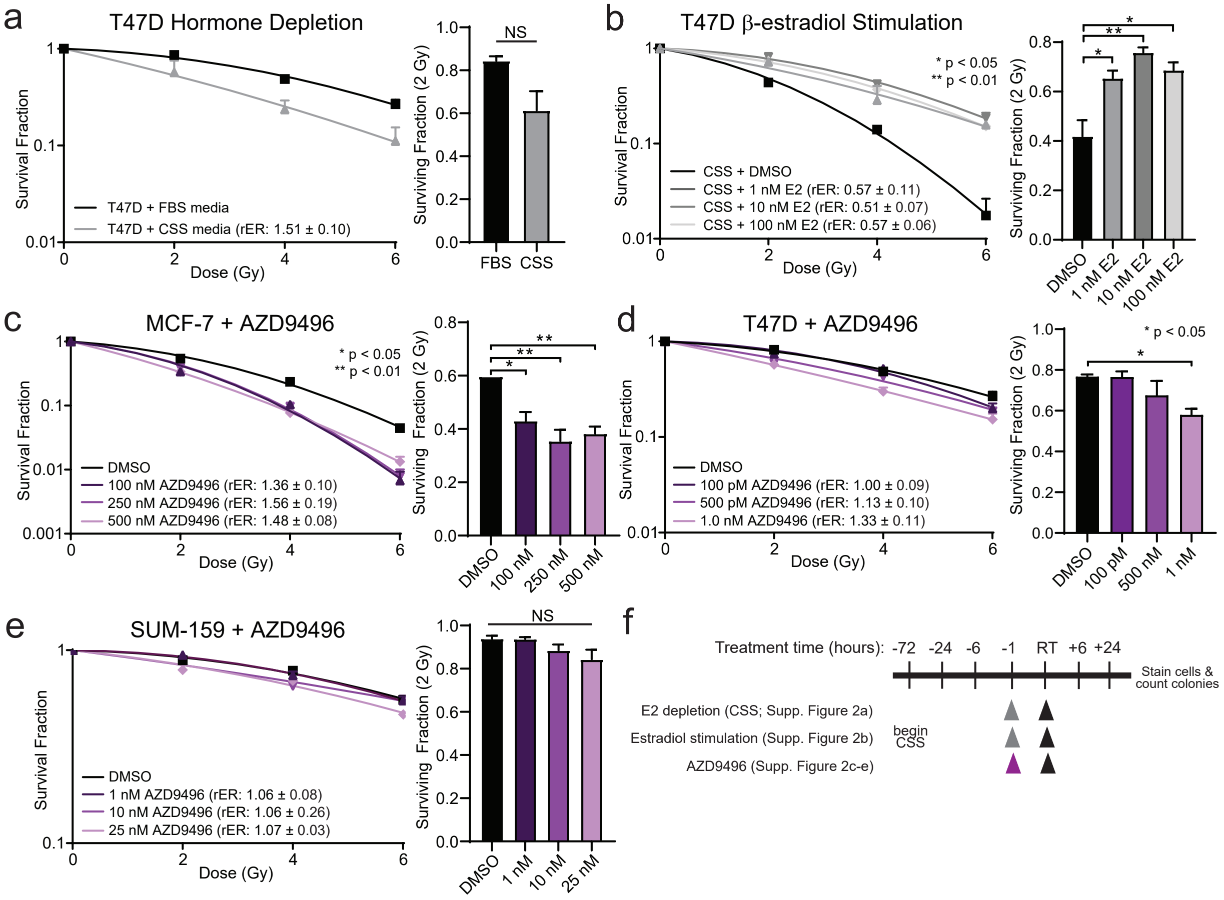

Supplementary Figure 2: **Radiosensitization with anti-estrogen therapies.** Clonogenic survival assays were performed in T47D cells pretreated with CSS for 1-hour compared to FBS-treated cells (**a**), or T47D cells pretreated for 3 days with CSS before stimulation with β-estradiol (**b**). Clonogenic survival assays were performed with a 1-hour pretreatment of AZD9496 in ER+ MCF-7 (**c**) or T47D (**d**), and ER-negative SUM-159 cells (**e**). A schematic of pretreatment times for clonogenic survival assays is shown (**f**). Clonogenic data is from triplicate experiments (a, c-e) or duplicate experiments (b) and is graphed as mean ± SEM. (* $p < 0.05$; ** $p < 0.01$; NS = not significant)

Supplementary Figure 3

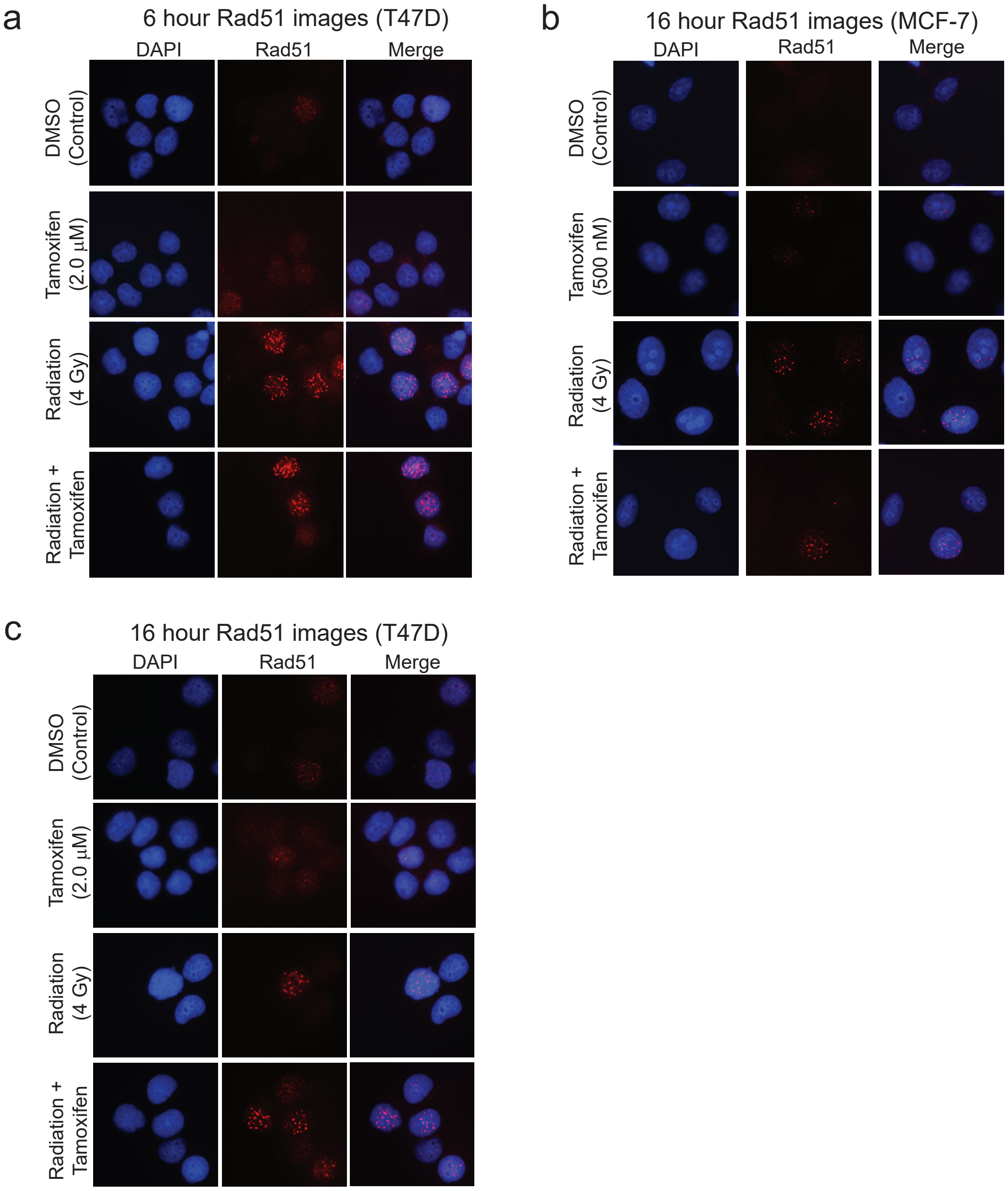

Supplementary Figure 3: Representative Rad51 foci images from immunofluorescence. Images of T47D cells ± tamoxifen ± 4 Gy RT at 6 hours post-RT are shown (**a**). Images for MCF-7 (**b**) or T47D (**c**) cells at 16 hours post-RT are shown for all treatment groups.

Supplementary Figure 4

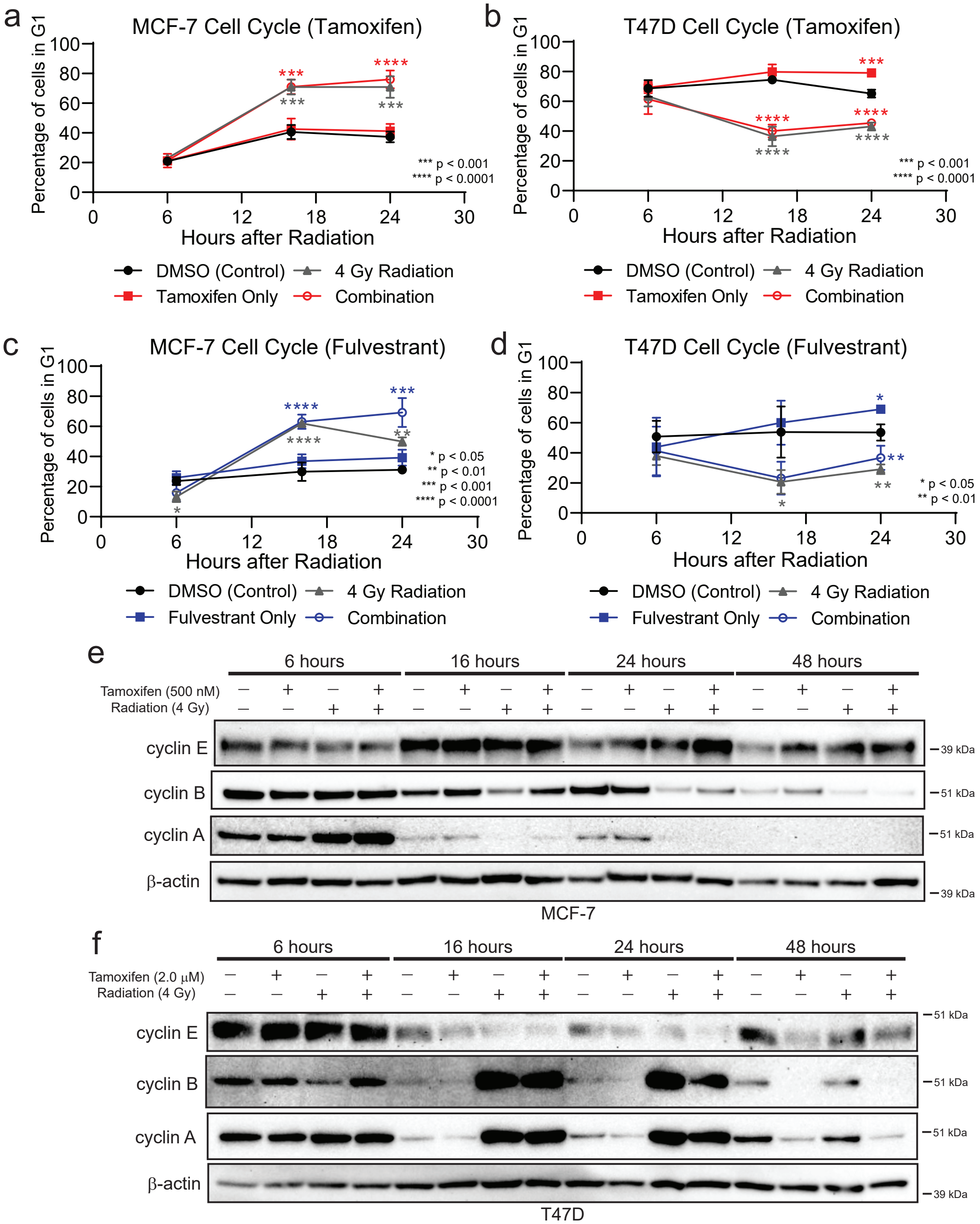

Supplementary Figure 4: **Radiation treatment promotes cell cycle arrest in ER+ MCF-7 and T47D cells.** Cells were pretreated for one hour with tamoxifen or fulvestrant prior to radiotherapy, fixed at the indicated timepoints (6-, 16-, 24-hours after radiation), and stained with propidium iodide before flow cytometry analysis. Gates were set to sort for spherical cells and remove doublets. Cell cycle progression was then assessed using a model fit based on propidium iodide staining. MCF-7 cells were treated with radiation ± tamoxifen (**a**). T47D cells were treated with radiation ± tamoxifen (**b**). Similarly, MCF-7 (**c**) or T47D (**d**) cells were treated with radiation ± fulvestrant. Expression of cyclins A, B, and E were assessed by western blot in MCF-7 (**e**) and T47D (**f**) cells treated with tamoxifen ± radiation. Data from triplicate experiments are graphed as mean ± SD. Western blots are representative of triplicate experiments. (* $p < 0.05$; ** $p < 0.01$; *** $p < 0.001$; **** $p < 0.0001$)

Supplementary Figure 5

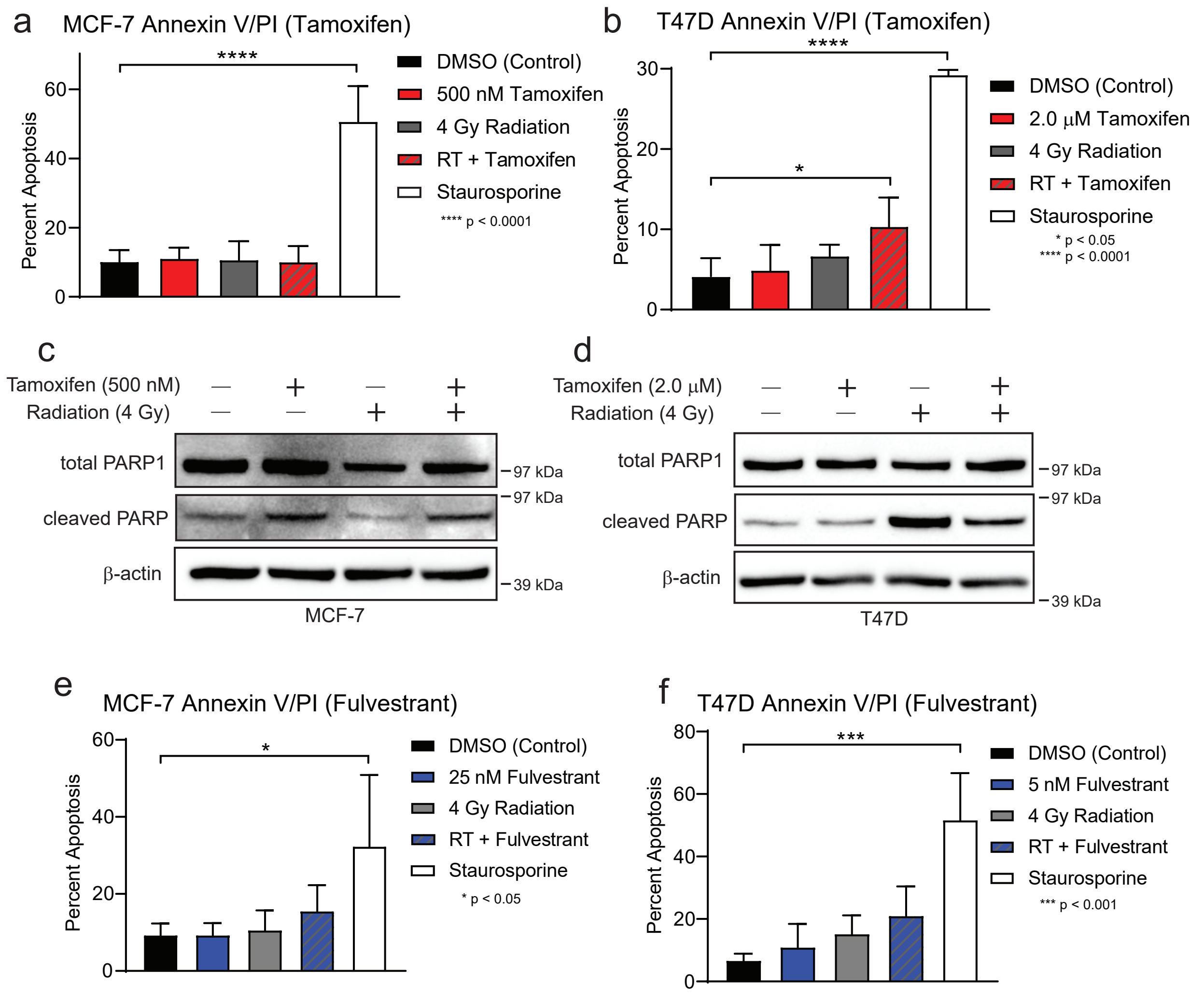

Supplementary Figure 5: Endocrine therapy treatment with radiation does not induce apoptosis in ER+ cells. Annexin V/PI staining was measured via flow cytometry to assess apoptotic cells (total cells undergoing early and late apoptosis); staurosporine (500 nM) was used as a positive control. Gates were set using cells unstained for annexin V, or propidium iodide, or cells stained with either annexin V or PI alone. Spherical cells were then selected, and cells with high Annexin V were categorized as undergoing early or late apoptosis, respectively, depending on the level of PI staining. MCF-7 cells were treated with tamoxifen (500 nM), 4 Gy RT, or combination treatment (**a**). T47D cells were treated with tamoxifen (2 μM), 4 Gy RT, or combination treatment (**b**). Levels of cleaved or total PARP1 were assessed by western blot in MCF-7 (**c**) and T47D (**d**) cells 48-hours after RT. Treatment with fulvestrant was also assessed by flow cytometry in MCF-7 (25 nM fulvestrant, **e**) and T47D (5 nM fulvestrant, **f**) cells. Data from triplicate experiments are graphed as mean ± SD. (* $p < 0.01$; *** $p < 0.001$; **** $p < 0.0001$)

Supplementary Figure 6

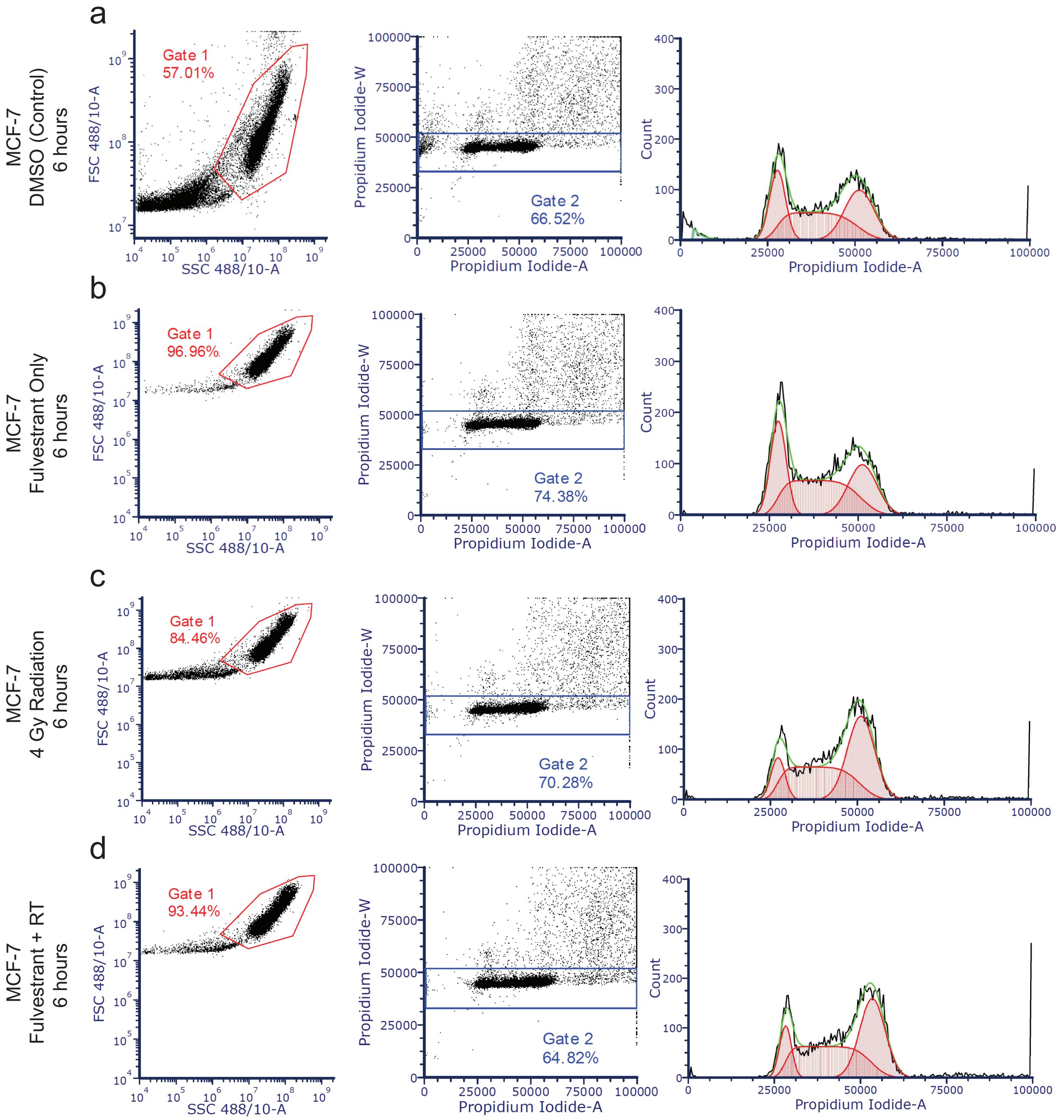

Supplementary Figure 6: Representative gating for PI staining of cell cycle by flow cytometry. Gating of MCF-7 cells stained for PI after 6-hour treatment with (a) DMSO (Control), (b) fulvestrant, (c) radiation, or (d) fulvestrant + RT. Gates were used to select for live cells, and analysis software was used to determine the distribution of cells in each phase of the cell cycle. The percentage of cells in G1 phase is indicated over time (6-24 hours) in Supplementary Figure 4.

Supplementary Figure 7

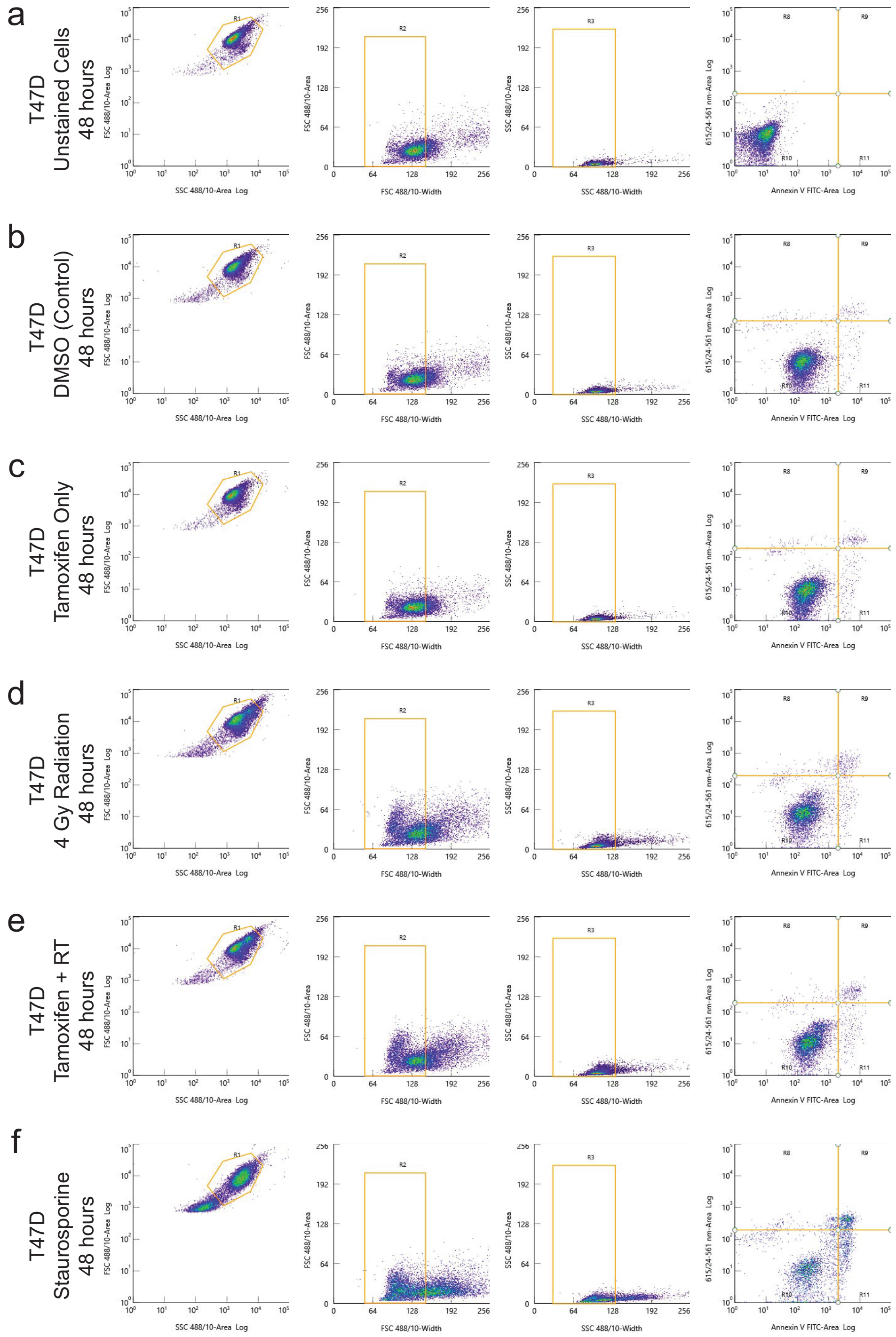

Supplementary Figure 7: Representative gating for AnnexinV/PI staining of apoptosis by flow cytometry. Gating of (a) unstained T47D cells or T47D cells stained for AnnexinV/PI after 48-hour treatment with (b) DMSO (Control), (c) tamoxifen, (d) radiation, (e) tamoxifen + RT, or (f) staurosporine as a positive control, are shown. Gates were used to select for intact cells, and cells positive for Annexin V undergoing early apoptosis (quadrant R11) or late apoptosis (quadrant R9) were combined to indicate the total percentage of cells undergoing apoptosis as indicated in Supplementary Figure 5.

Figure 3E (Full blot)

Rad51 (MCF-7)

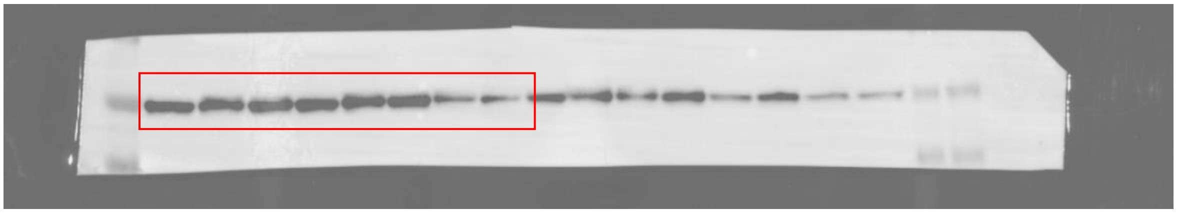

The red box highlights the area used in the figure. The additional samples were from an extended time course.

β-actin (MCF-7)

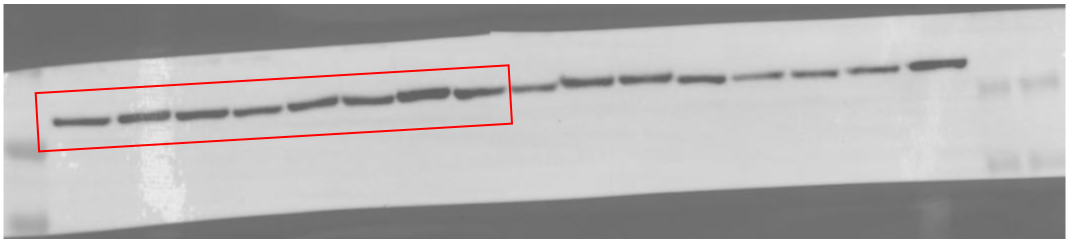

The red box highlights the area used in the figure. The additional samples were from an extended time course.

Rad51 (T47D)

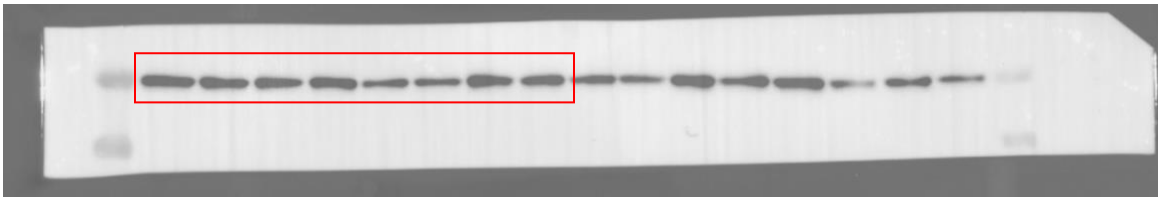

The red box highlights the area used in the figure. The additional samples were from an extended time course.

β-actin (T47D)

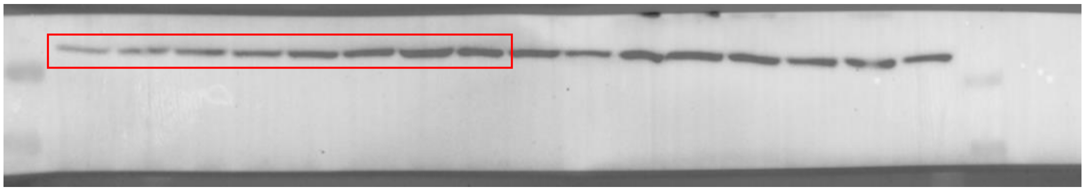

The red box highlights the area used in the figure. The additional samples were from an extended time course.

Supplementary Figure 1E (Full blot)

ERα (MCF-7)

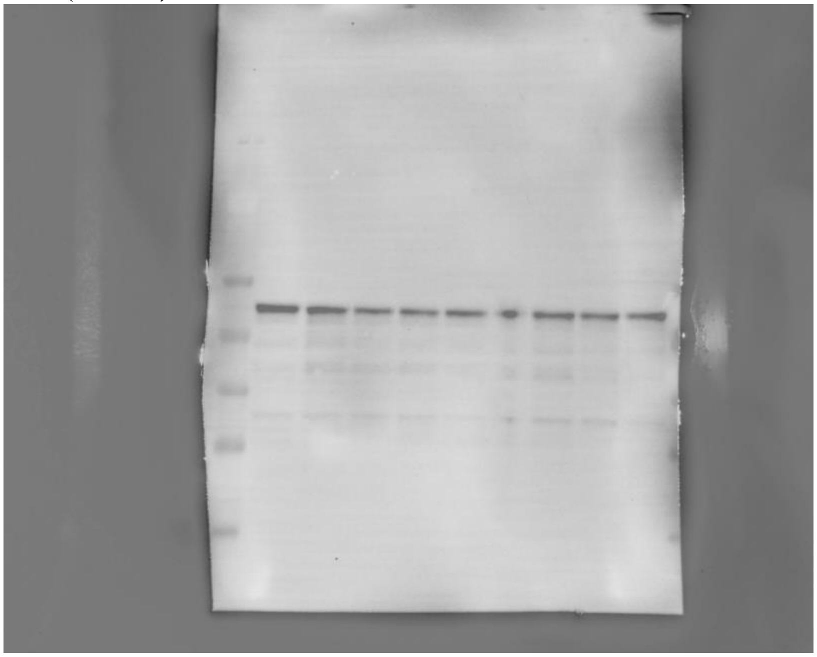

β-actin (MCF-7)

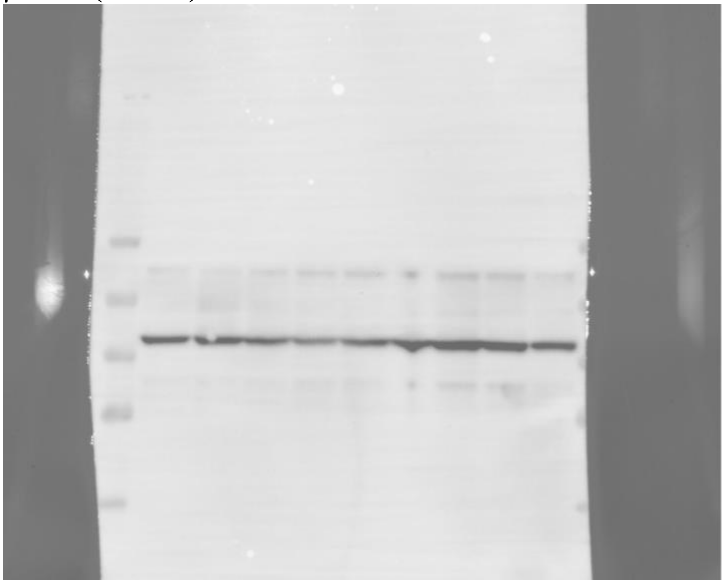

Supplementary Figure 1F (Full blot)
ERα (T47D)

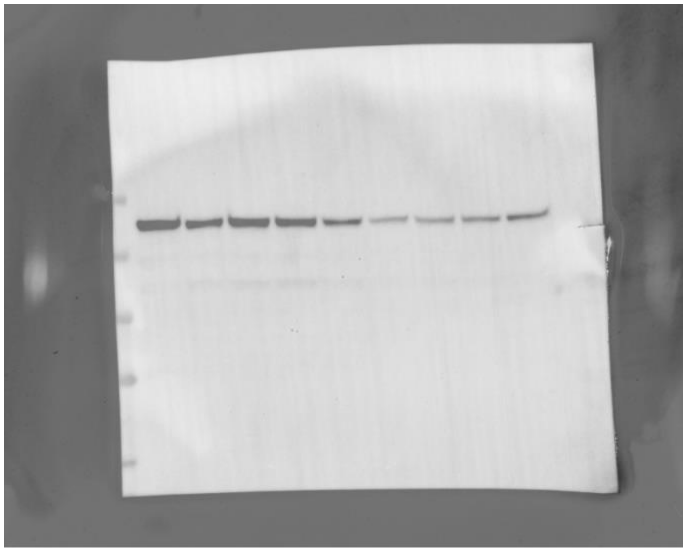

β-actin (T47D)

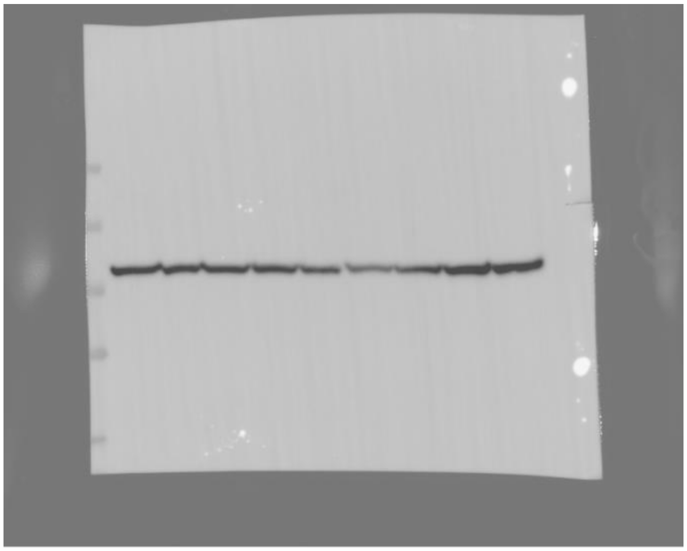

Supplementary Figure 4E (Full blot)

Cyclin E (MCF-7)

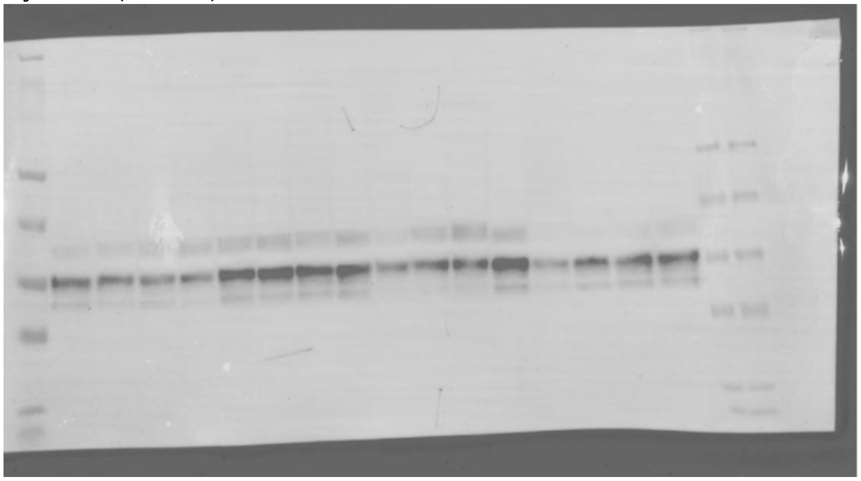

Cyclin B (MCF-7)

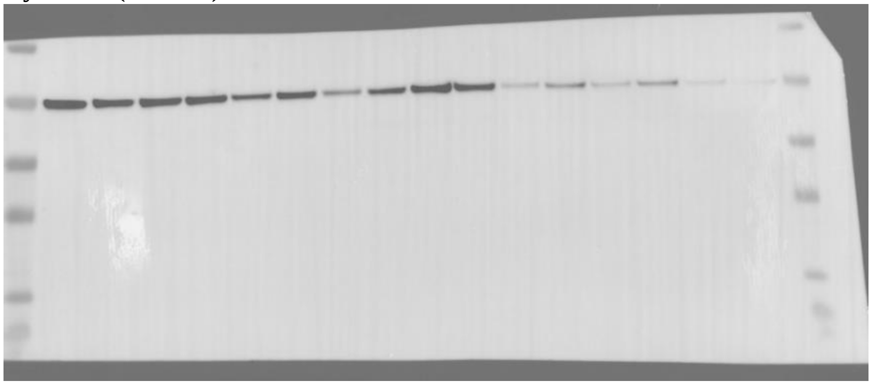

Cyclin A (MCF-7)

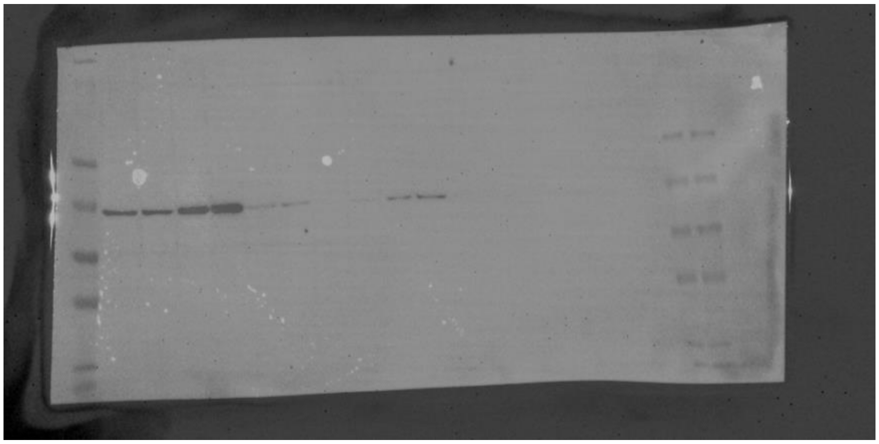

β-actin (MCF-7)

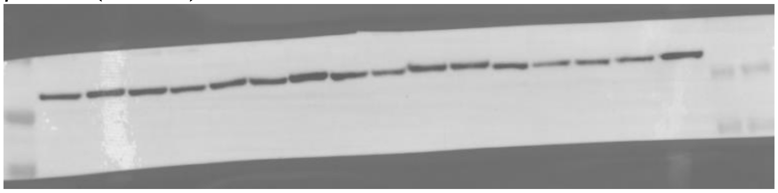

Supplementary Figure 4F (Full blot)

Cyclin E (T47D)

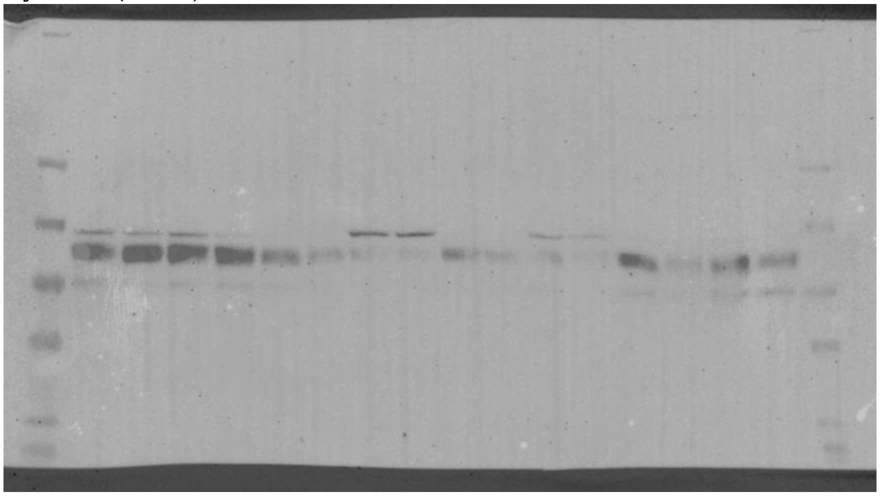

Cyclin B (T47D)

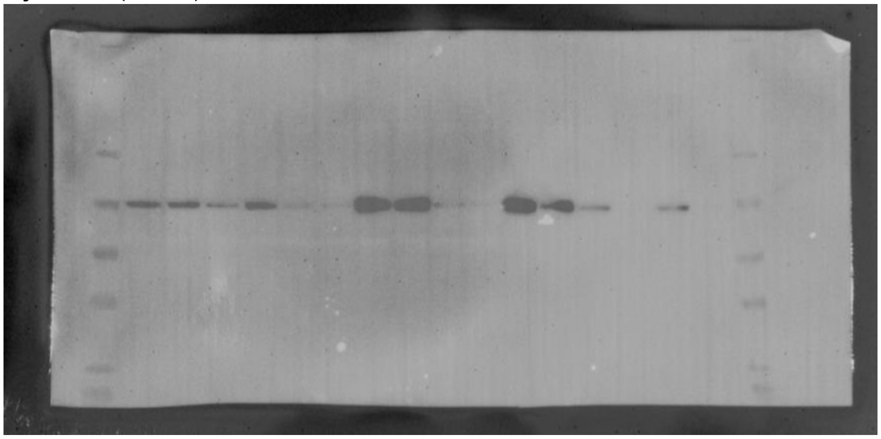

Cyclin A (T47D

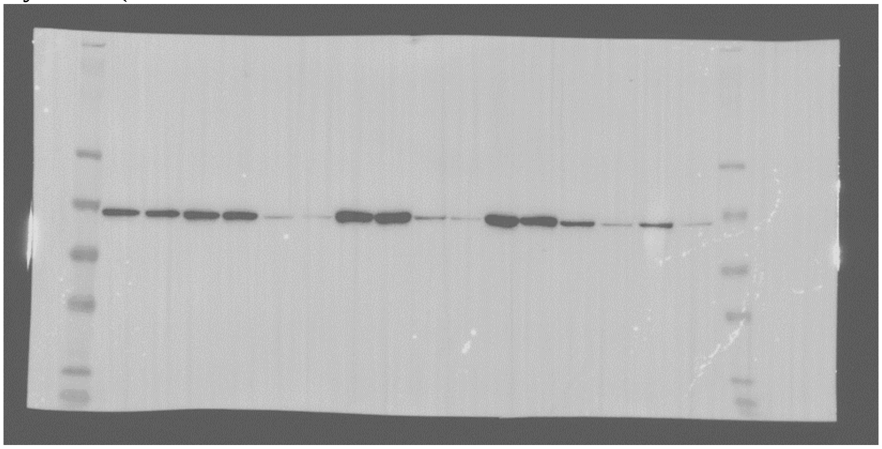

β-actin (T47D)

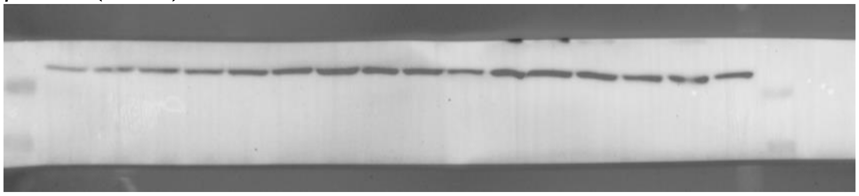

Supplementary Figure 5C (Full blot)

Total PARP1 & Cleaved PARP (MCF-7)

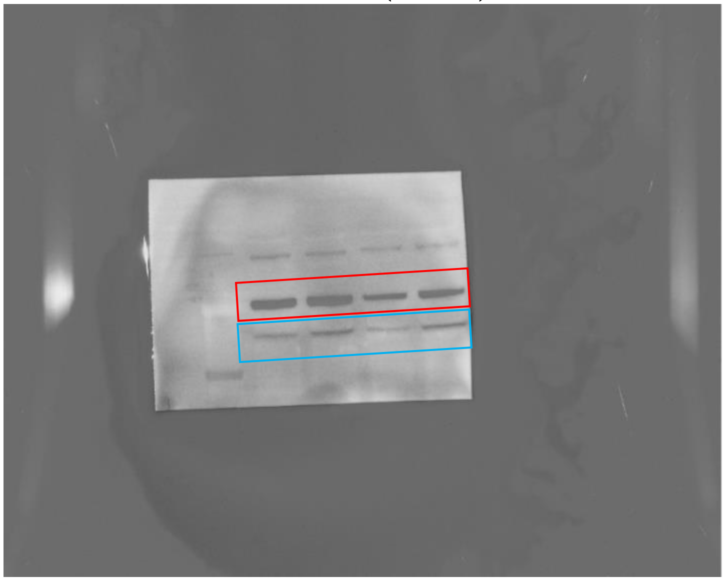

The red box outlines total PARP1 protein. The blue box outlines cleaved PARP.

β-actin (MCF-7)

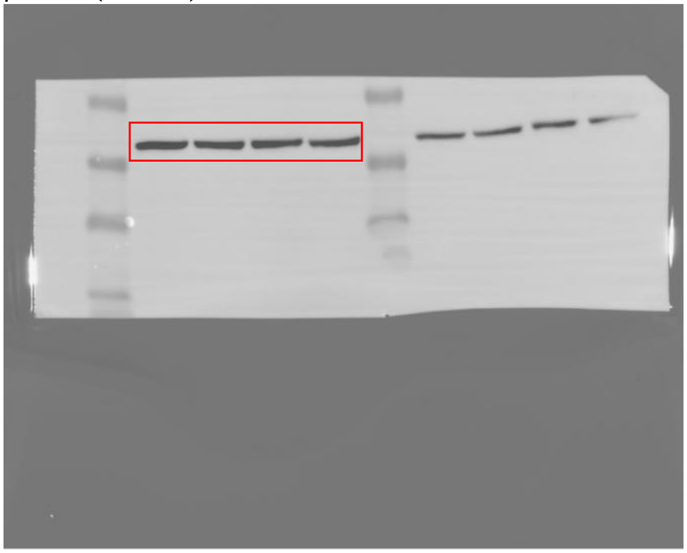

The red box highlights the area used in the figure. The samples on the right side of the blot were from another cell line (T47D).

Supplementary Figure 5C (Full blot):

Total PARP1 & Cleaved PARP (T47D)

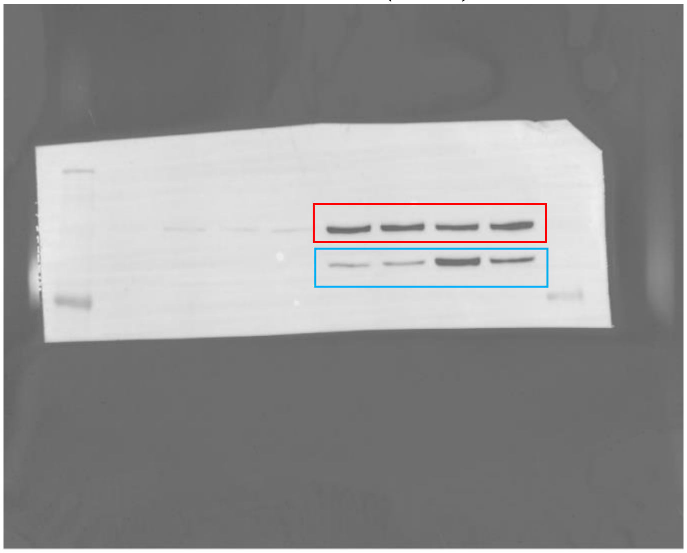

The red box outlines total PARP1 protein. The blue box outlines cleaved PARP.

β-actin (T47D)

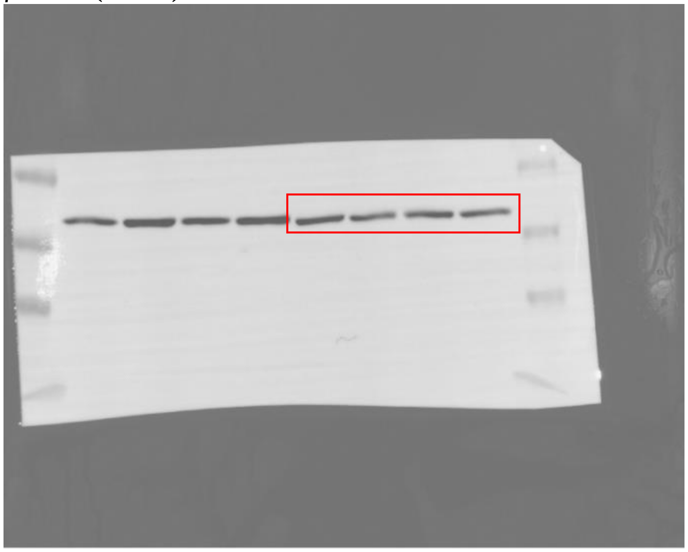

The red box highlights the area used in the figure. The samples on the left side of the blot were from another cell line (T47D).
